# A Risk Prediction Model for Breast Cancer Based on Immune Genes Related to Early Growth Response Proteins Family

**DOI:** 10.3389/fmolb.2020.616547

**Published:** 2021-02-03

**Authors:** Xin Zhou, Fang-yuan Zhang, Yan Liu, Dong-xin Wei

**Affiliations:** Department of Breast Surgery, Zibo Maternal and Child Health Hospital, Zibo, China

**Keywords:** EGR family, breast cancer, DNA methylation, genetic alteration, immune cells, prognosis, nomogram

## Abstract

Early growth response proteins (EGRs), a transcriptional regulatory family comprised of EGR1, EGR2, EGR3, and EGR 4, are reportedly involved in a vast array of functions. However, EGRs, as a whole, are rarely studied in breast cancer cases. This research was performed based on public datasets. The results demonstrated that, except EGR4, the other EGRs were differentially expressed genes in breast cancer. Subsequently, this study determined the prognosis significance of the EGR family, higher expression levels of EGRs indicating better overall survival (OS) and disease-free survival (DFS), except EGR4. So we attempted to explore the potential mechanism behind the prognostic value of EGRs. At the DNA level, however, neither DNA methylation status nor genetic alterations of EGRs contributed to the prognosis significance. Kyoto Encyclopedia of Genes and Genomes (KEGG) pathway analysis revealed that EGRs were involved in several immune-related functions. Afterward, we assessed the correlation between EGRs and the immune system before establishing a risk prediction model with a 14-gene immune signature associated with EGRs, a prognostic nomogram predicting individuals’ 1-, 3-, and 5-year survival probabilities. The risk score was an independent prognosis predictor in the breast cancer cohorts. This study evidenced EGRs’ significance for tumor immunity, demonstrating that the EGR family may be a potential immunotherapeutic target for breast cancer. The 14-gene immune signature is a promising prognostic biomarker in breast cancer.

## Introduction

In cancer research, the tumor microenvironment has become a promising field with fast development. Almost all types of carcinoma are divided into different subtypes by immune genes and tumor-infiltrating immune cells. Significant breakthroughs of immunotherapy have been demonstrated in some cancer categories, such as melanoma and non-small-cell lung cancer (NSCLC). Immunotherapy works by enhancing the immune machinery of the patient to identify cancer as a foreign antigen, which would destroy the cancer cells. Its antitumor function includes presenting antigen to T cells (largely through dendritic cells), effector T cells’ trafficking and infiltrating process into the bed of tumor, then identifying the T cells’ infiltration, and eradicating tumor cells (Tokumaru et al., 2020). Though breast cancer was regarded as an “immune cold tumor,” an increasing number of reports about this field in breast cancer have emerged over the past few years. Patients with certain subtypes of breast cancer would benefit from immunotherapy (Naik et al., 2019). However, the full potential benefit of immunotherapy has yet to be proved in breast cancer (Mina et al., 2019). Novel targets and mechanisms require further discovery.

Early growth response proteins (EGRs) refer to a transcriptional regulatory family consisting of four members, including EGR1, EGR2, EGR3, and EGR 4. All the EGRs contain three cyc2-His2 zine fingers (Poirier et al., 2008) and are involved in extensive functions (Taefehshokr et al., 2017). Specific to the relationship between EGRs and the immune system, EGR1 helps positively select CD4 and CD8 single-positive cells (Bettini et al., 2002), EGR2 and EGR3 act as unique regulators in the immune system (Larsson et al., 1998; O’Donovan et al., 1999), and EGR4 is involved Th1 differentiation by suppressing Ca 2+ signals in vivo (Mookerjee-Basu et al., 2020). However, the precise correlation between the immune system and the whole EGR family members remains unclear.

Yuchang Fei et al. investigated the prognostic value of the EGR family in breast cancer by using data mining methods but had not revealed the potential mechanism behind that (Fei et al., 2019). Here, the present study assessed the prognosis significance of each EGR family member, based on which we further explored the potential mechanism and revealed their correlations with tumor-infiltrating immune cells in breast cancer. Finally, we generated a 14-gene prognostic immune signature using EGRs-associated immune genes, followed by the construction of a nomogram by combining the immune signature and other clinical features.

## Materials and Methods

### Data Acquisition

Datasets of patients with breast invasive carcinoma (BRCA) were extracted from The Cancer Genome Atlas (TCGA) Pan-cancer datasets, which were downloaded from the UCSC Xena database (http://xena.ucsc.edu/) (Goldman et al., 2020), including 1098 cancerous and 113 normal tissues. All the RNA-seq data (level 3) were normalized as fragments per kilobase of transcript per million mapped reads.

### Expression Analysis

Inside the Tumor Immune Estimation Resource (TIMER2.0) algorithm database (https://cistrome.shinyapps.io/timer/) (Li et al., 2020), gene expression levels of EGRs in a range of cancers were found. Expressions of EGRs in breast cancer were assessed via the GEPIA2 database, which is a web tool for wide-ranged expression profiling and interactive analysis with data from the TCGA database and the Genotype-Tissue Expression (GTEx) database (http://gepia.cancer-pku.cn/) (Tang et al., 2019). Then we investigated the correlation patterns among EGRs, as well as the expression profile according to a range of clinicopathological characteristics of breast cancer, including hormonal receptors status, HER2 status, age, nodal status, Nottingham prognostic index (NPI), and Scarff-Bloom-Richardson (SBR) grade by using all RNA-seq data from bc-GenExMiner v4.5 database, which is a user-friendly online mining platform with published annotated data of breast cancer (http://bcgenex.centregauducheau.fr) (Jézéquel et al., 2012, 2013). *p* < 0.05 was considered statistically significant.

### Prognostic Analysis

We determined the prognosis significance of the EGR family in breast cancer by using all DNA microarray data (Affymetrix and METABRIC) (*n* = 10001) and verified it with all RNA-seq data (TCGA and SCAN-B) (*n* = 4712) via bc-GenExMiner v4.5 database. Subtype analyses were also performed by using all DNA microarray data. *p* < 0.05 was considered statistically significant.

### Gene Alteration Analysis

Gene alterations of EGRs were explored by using data from the Breast Invasive Carcinoma (TCGA, Firehose Legacy) samples via the cBioPortal database, which is a portal for integrative analysis of cancer genomics and clinical profiles (Gao et al., 2013; Cerami et al., 2012). The tab OncoPrint overviews genetic variations per sample in the respective EGR family member. Relative linear copy number values of EGRs from the Breast Invasive Carcinoma (TCGA, Firehose Legacy) dataset were downloaded via the cBioPortal database and were used to perform subtype analysis. The tab Comparison shows the correlation between mRNA expressions of EGRs and copy number alterations. In the tab Survival, using the Kaplan–Meier analysis is capable of assessing the effect of the gene alterations on overall survival (OS) and disease-free survival (DFS). *p* < 0.05 was considered statistically significant.

### DNA Methylation Analysis

In this part, the DNA methylation status of EGRs in normal breast tissues and malignant counterparts was initially identified by using the DNA Methylation Interactive Visualization Database (DNMIVD), which is an online platform for DNA methylation interactive visualization (http://119.3.41.228/dnmivd/index/)(Ding et al., 2019, 2020a, 2020b). DNA methylation (HM450) data of EGRs from the Breast Invasive Carcinoma (TCGA, Firehose Legacy) dataset were downloaded via the cBioPortal database to perform subtype analysis. We also analyzed the prognosis significance of the DNA methylation status of the EGR family via the DNMIVD. *p* < 0.05 was considered statistically significant.

### Correlation and Functional Analyses of Early Growth Response Proteins

We used three databases, including bc-GenExMiner v4.5, GeneMANIA, which is an online web tool that can identify the most related genes to a query gene set using a guilt-by-association approach and conduct gene function prediction (http://genemania.org/) (Montojo et al., 2014), and STRING, which is an online tool aiming to collect, score, and integrate all publicly available sources of protein-protein interaction information, and to complement these with computational predictions (https://string-db.org/) (Szklarczyk et al., 2019). This study analyzed the correlation among all EGRs in bc-GenExMiner v4.5. Subsequently, by using GeneMANIA and STRING, the interactions of EGRs at the gene and protein level were identified. Afterward, we obtained the top 20 genes associated with EGRs; together with EGRs, we performed functional analyses, including Gene Ontology (GO) enrichment analysis and Kyoto Encyclopedia of Genes and Genomes (KEGG) pathway enrichment analysis, by using the STRING database. The GO enrichment analysis includes biological processes (BP), cellular components (CC), and molecular function (MF). False discovery rate (FDR) < 0.05 was considered statistically significant.

### Correlation Between Early Growth Response Proteins and Tumor Immune Infiltration

Related infiltration and activity levels for 28 immune cell types, obtained from published signature gene lists across all tumor and normal samples, were quantified using the ssGSEA in R package GSVA (Hänzelmann et al., 2013). The signatures used in this study include activated B cell (Act B), activated CD4 T cell (Act CD4), activated CD8 T cell (Act CD8), activated dendritic cell (Act DC), CD56 bright natural killer cell (CD56bright), CD56 dim natural killer cell (CD56dim), central memory CD4 T cell (Tcm CD4), central memory CD8 T cell (Tcm CD8), effector memory CD4 T cell (Tem CD4), effector memory CD8 T cell (Tem CD8), eosinophil, gamma delta T cell (Tgd), immature B cell (Imm B), immature dendritic cell (iDC), macrophage, mast cell, myeloid-derived suppressor cells (MDSC), memory B cell (Mem B), monocyte, natural killer cell (NK), natural killer T cell (NKT), neutrophil, plasmacytoid dendritic cell (pDC), regulatory T cell (Treg), T follicular helper cell (Tfh), type 1 T helper cell (Th1), type 2 T helper cell (Th2), and type 17 T helper cell (Th17). The ssGSEA scores for each immune cell type were standardized. Then by using the BRCA data from TCGA, we investigated the different levels of tumor-infiltrating immune cells (TIICs) between BRCA and normal tissues, influences of EGRs on TIICs, and the correlations between EGRs and TIICs. *p* < 0.05 was considered statistically significant.

### Construction of Risk Prediction Model

In this part, we carried on analyses by using R version 4.2 (R Foundation for Statistical Computing, Vienna, Austria). Information about genes related to the immune system, including immunoinhibitors, immunostimulators, MHCs, chemokines, and receptors, is available from the TISIDB database (http://cis.hku.hk/TISIDB/index.php), which is a web portal for tumor and immune system interaction (Ru et al., 2019). Heatmaps of correlations between the mentioned genes and EGRs are available via the TISIDB database; we replotted the heatmaps with R. Then we conducted univariate Cox regression analysis with immune genes associated with all EGRs (Spearman correlation test, *p* < 0.05), generated a 14-gene prognostic signature, and conducted multivariate Cox regression analysis with these signature genes. Then risk score was generated: risk score = *β*_1_x_1_+*β*_2_x_2_+…+*β*_i_x_i_. In this formula, xi was the expression level of each gene, while *β*_i_ is the risk coefficient of each gene derived from the Cox model (Aguirre-Gamboa et al., 2013). Kaplan–Meier survival curve, log-rank test, and the time-dependent receiver operating characteristic (ROC) curves were adopted to appraise the association of the gene signature with overall survival. Lastly, we constructed a prognostic nomogram in BRCA for anticipating the individuals’ survival probability by weighing risk score, age, and stage. The concordance index (C-index), calibration curves, and time-dependent ROC curves were used for the evaluation of the risk prognostic model. *p* < 0.05 was considered statistically significant. We also performed functional analysis on the 14 genes via the STRING database.

### Validation of the Risk Prediction Model Using the METABRIC Cohort

Normalized gene expression data of METABRIC, the largest available breast cancer data cohort, were downloaded from the cBioPortal database. METABRIC, short for Molecular Taxonomy of Breast Cancer International Consortium, consists of 1905 breast tumor samples with both genotype and gene expression data.

### Statistics

Data download from TGCA were merged and conducted by R version 4.2 (R Foundation for Statistical Computing, Vienna, Austria). Wilcoxon test was used to compare two cohorts’ continuous variables. Univariate Cox analysis was used to generate a 14-gene signature associated with OS in breast cancer. Nomogram based on multivariate Cox analysis was employed to construct a risk prediction model. The strength of the correlation was determined using the following guide for the absolute value: 0.00–0.29 (weak), 0.30–0.59 (moderate), 0.60–0.79(strong), and 0.80–1.0 (very strong) (Pan et al., 2019). All the thresholds of statistical significance were set as *p* < 0.05.

## Results

### Aberrant Expressions of Early Growth Response Proteins in Breast Invasive Carcinoma Patients

**FIGURE 1:**
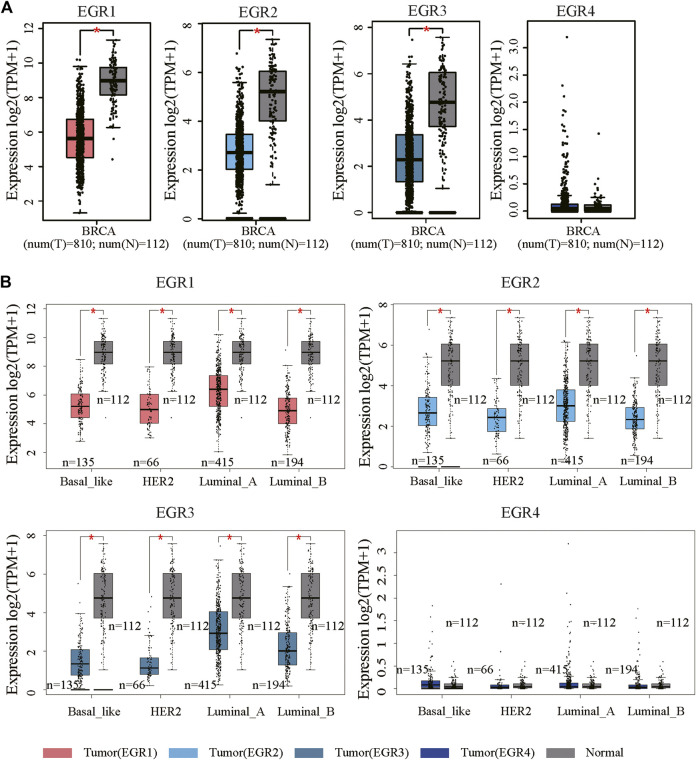
mRNA expression levels of EGRs in BRCA. **(A)** The mRNA expression levels of EGRs in breast cancer and normal tissues (*p < 0.05). **(B)** Comparison of EGRs mRNA levels between different subtypes of BRCA and normal tissues (*p < 0.05).

**FIGURE 2:**
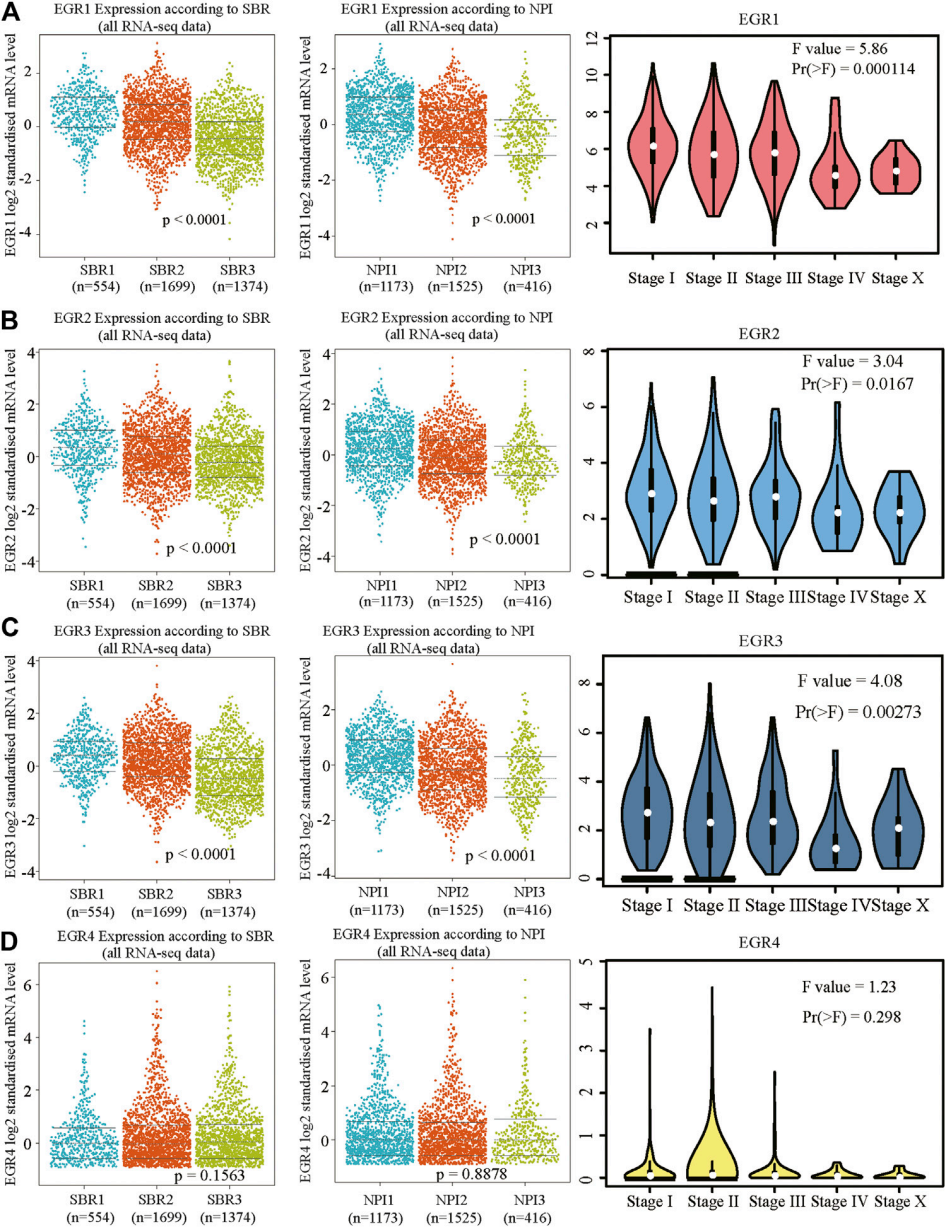
Relationship between mRNA levels of EGRs and SBR, NPI, or stage in BRCA. Beeswarm plots of EGRs expressions according to SBR, NPI, and stage. **(A)** EGR1, **(B)** EGR2, **(C)** EGR3, and **(D)** EGR4.

We first determined EGRs expression levels in different tumor types and normal counterparts using the TIMER2.0 database. Accordingly, EGR1, EGR2, and EGR3 were differentially expressed genes (DEGs) in several cancers, such as bladder urothelial carcinoma (BLCA), BRCA, and lung adenocarcinoma (LUAD). EGR4 was a DEG in colon adenocarcinoma (COAD), glioblastoma multiforme (GBM), etc. (Supplementary Figure S1). Then we explored the transcriptional levels of 4 EGR family members in BRCA and normal breast tissues with GEPIA2. As indicated by the results, the transcriptional levels of EGR1, EGR2, and EGR3 were significantly reduced in patients with BRCA (*p* < 0.05), whereas the expression level of EGR4 was very low in both breast cancer and normal breast tissues (Figure 1A). Hence, in some way, EGR4 can be regarded as a nonexpressing gene in both breast cancer and benign counterparts. Afterward, in subtype analysis, it suggested that EGR1, EGR2, and EGR3 were downregulated in all 4 subtypes of BRCA, including Luminal A, Luminal B, Her2, and Base-like subtypes (*p* < 0.05) (Figure 1B). Besides, we explored the mRNA expressions of EGRs according to different clinicopathological characteristics of BRCA. As revealed from the results, lower EGR1, EGR2, and EGR3 levels displayed a relationship to higher SBR grade, NPI, and tumor stage, respectively (*p* < 0.0001) (Figures 2A–C). However, EGR4 was not associated with SBR, NPI, or tumor stage (Figure 2D). For other clinical indicators, EGR1 displayed an association to estrogen receptor (ER) status, progesterone receptor (PR) status, human epidermal growth factor receptor-2 (HER2) status, and nodal status (N); EGR2 displayed an association to PR status, HER2 status, and age; EGR3 displayed an association to ER status, PR status, HER2 status, and age; EGR4 displayed an association to ER status, PR status, and HER2 status (Supplementary Figure S2).

### Prognosis Significance of Early Growth Response Proteins in Breast Invasive Carcinoma Patients

**FIGURE 3:**
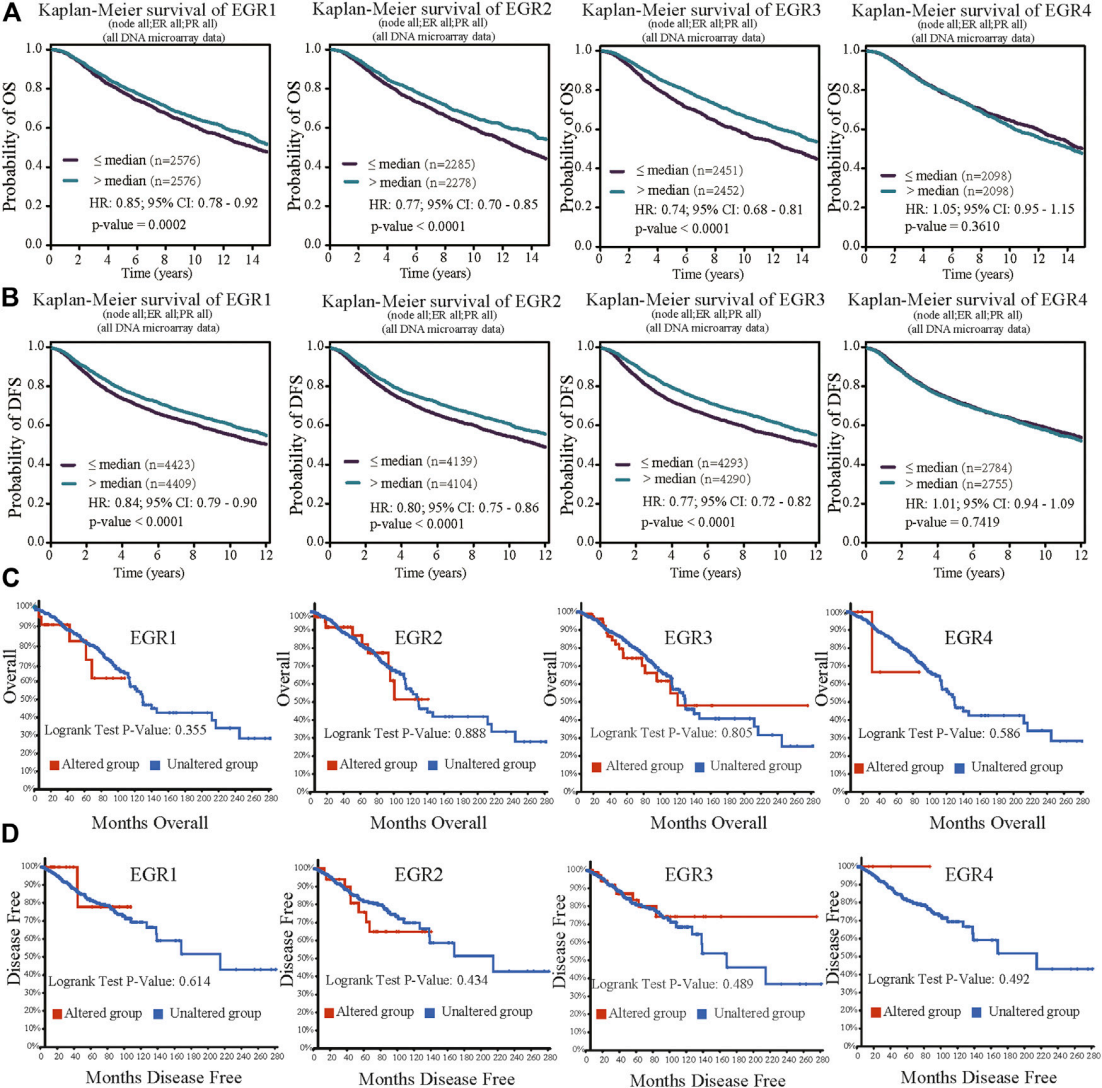
Prognostic value of EGRs mRNA expressions (all DNA microarray data from bc-GenExMiner v4.5) and genetic alterations. **(A)** Except EGR4, other members of the EGR family were associated with OS, higher levels indicating better outcome (EGR1, *p* = 0.0002; EGR2, *p* < 0.0001; EGR3, *p* < 0.0001; EGR4, *p* = 0.3610). **(B)** Except EGR4, other members of the EGR family were associated with DFS, higher levels indicating better outcome (EGR1, *p* < 0.0001; EGR2, *p* < 0.0001; EGR3, *p* < 0.0001; EGR4, *p* = 0.7419). **(C)** No correlations were found between genetic alterations of EGRs and OS. **(D)** No correlations were found between genetic alterations of EGRs and DFS.

Within the bc-GenExMiner v4.5 database, by using all DNA microarray data (*n* = 10001), we found significant prognostic values of EGR1, EGR2, and EGR3, patients with higher expressions of these genes showed favorable OS (HR = 0.85, *p* = 0.0002; HR = 0.77, *p* < 0.0001; HR = 0.77, *p* < 0.0001) and DFS (HR = 0.76, *p* = 0.0011; HR = 0.80, *p* < 0.0001; HR = 0.77, *p* < 0.0001) separately. EGR4 was not a prognostic maker in BRCA (HR = 1.05, *p* =0.3610; HR = 0.94, *p* = 0.1745) (Figures 3A,B). Then we verified the prognosis significance of EGRs with all RNA-seq data via the bc-GenExMiner v4.5 database (Supplementary Figure S3). Furthermore, we analyzed the prognostic significance of the EGR family in different PAM50 subtypes of BRCA using all DNA microarray data. The detailed results were summarized in Table 1 and survival curves of significant results were demonstrated in Supplementary Figure S4. EGR1 and EGR4 were not associated with OS or DFS in all four subtypes (*p* > 0.05). Higher level of EGR2 predicted better OS (HR = 0.80, *p* = 0.0253) and DFS (HR = 0.82, *p* = 0.0074) in Luminal A type and better OS (HR = 0.76, *p* = 0.0128) and DFS (HR = 0.85, *p* = 0.0460) in basal type and showed a relationship to better DFS (HR = 0.78, *p* = 0.0040) in HER2+ type. Elevated EGR3 level significantly correlated with better OS (HR = 0.82, *p* = 0.0370) and DFS (HR = 0.81, *p* = 0.0038) in Luminal A type.

**TABLE 1:** The correlation between EGRs and survival outcomes in different PAM50 subtypes of BRCA (all DNA microarray data).

Gene symbol	Survival outcome	Luminal A		Luminal B		HER2+		Basal	
**EGR1**		HR (95% CI) p-value		HR (95% CI) p-value		HR (95% CI) p-value		HR (95% CI) p-value	
	OS	0.93 (0.77–1.09)	0.4446	0.99(0.82–1.20)	0.9482	1.06(0.86–1.30)	0.5779	0.93(0.76–1.14)	0.4661
	DFS	0.91(0.79–1.05)	0.1989	0.95(0.83–1.10)	0.5169	0.99(0.84–1.16)	0.8694	0.90(0.77–1.05)	0.1715
**EGR2**	OS	0.80(0.66–0.97)	**0.0253**	0.92(0.76–1.12)	0.3995	0.81(0.66–1.01)	0.0571	0.76(0.61–0.94)	**0.0128**
	DFS	0.82(0.71–0.95)	**0.0074**	0.86(0.75–1.00)	0.0506	0.78(0.66–0.92)	**0.0040**	0.85(0.73–1.00)	**0.0460**
**EGR3**	OS	0.82(0.68–0.99)	**0.0370**	0.94(0.78–1.14)	0.5341	0.88(0.71–1.08)	0.2101	0.93(0.75–1.14)	0.4676
	DFS	0.81(0.70–0.93)	**0.0038**	0.90(0.78–1.04)	0.1669	0.88(0.75–1.04)	0.1246	0.95(0.81–1.11)	0.5180
**EGR4**	OS	1.04(0.86–1.27)	0.6722	1.03(0.84–1.25)	0.7925	0.99(0.80–1.22)	0.9367	1.02(0.82–1.27)	0.8589
	DFS	0.95(0.82–1.11)	0.5312	1.03(0.89–1.19)	0.7066	1.03(0.87–1.21)	0.7669	0.96(0.87–1.20)	0.7787

The significance of bold means *p*-values ≤ 0.05.

### Genetic Alterations of Early Growth Response Proteins in Breast Invasive Carcinoma Patients

We used the cBioPortal database to determine the type and frequency of EGRs genetic alterations in BRCA patients based on the data from the Breast Invasive Carcinoma (TCGA, Firehose Legacy). The result showed that the percentages of EGRs genetic alterations were 2.8% (31/1098) in EGR1 expression, 4% (45/1098) in EGR2 expression, 10% (105/1098) in EGR3 expression, and 0.5% (5/1098) in EGR4 expression (Supplementary Figure S5A). Next, we investigated the correlations between mRNA levels and gene alterations of EGRs. It showed moderate correlation between EGR3 mRNA expression level and genetic alteration (EGR3, Spearman = 0.32, *p* = 2.16e-27; Pearson = 0.31, *p* = 1.13e-25) and weak correlations between mRNA levels of other EGR family members and genetic alterations (EGR1, Spearman = 0.09, *p* = 1.837e-3; Pearson = 0.08, *p* = 0.0118; EGR2, Spearman = 0.05, *p* = 0.0787; Pearson = 0.06, *p* = 0.0451; EGR4, Spearman = 0.17, *p* = 1.73e-8; Pearson = 0.17, *p* = 2.70e-8) (Supplementary Figure S5B). The copy number variation of EGR1 was associated with ER status, PR status, and TNBC status. The copy number variation of EGR3 was associated with PR status and HER2 status. The copy number variation of EGR4 was associated with ER status, PR status, HER2 status, and TNBC status (Supplementary Figure S6). Then in survival analysis, genetic alterations of all the EGR family members showed no correlations to OS or DFS (Figures 3C,D).

### DNA Methylation of Early Growth Response Proteins in Breast Invasive Carcinoma Patients

**FIGURE 4:**
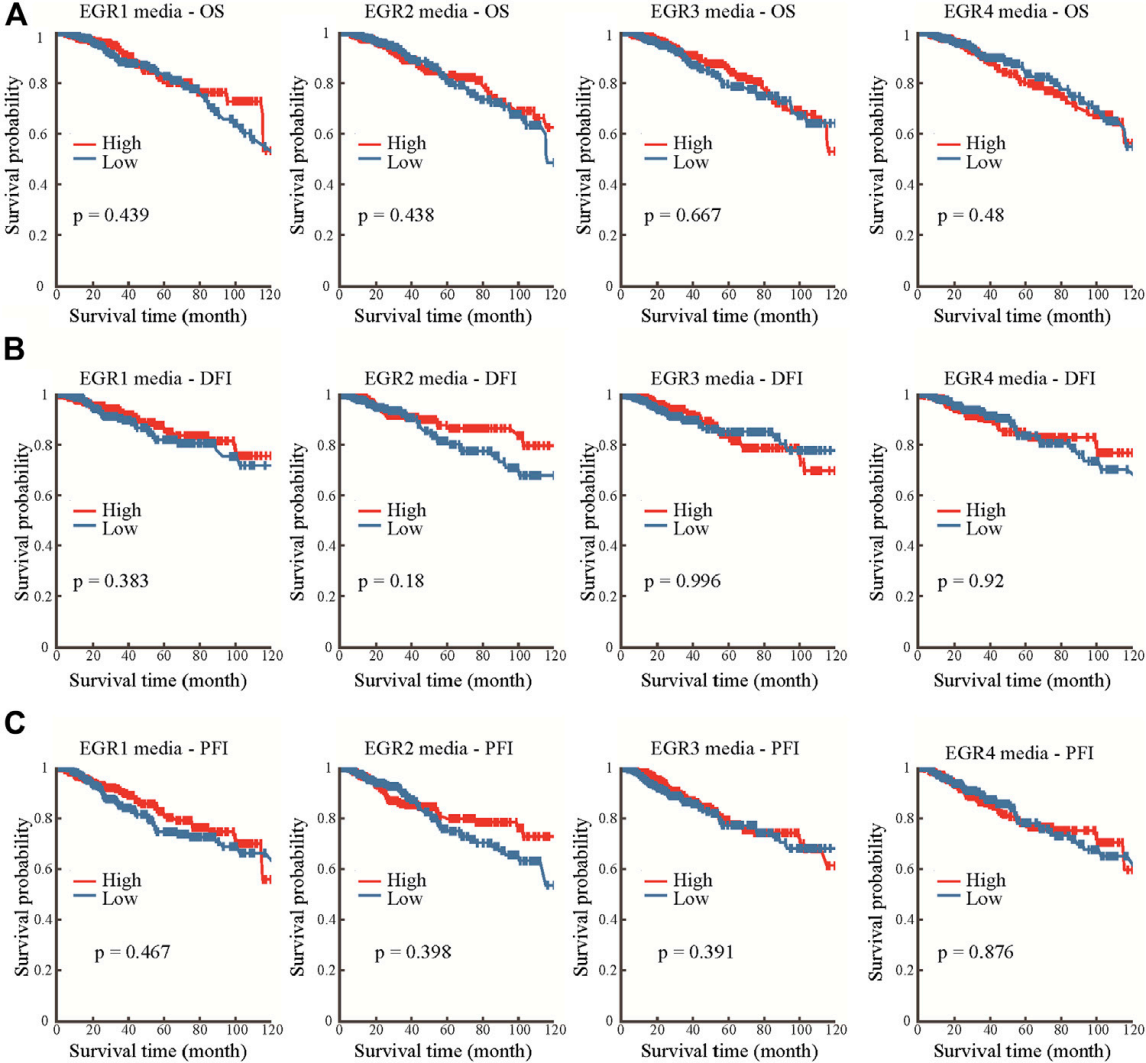
Prognostic value of the DNA methylation status of EGRs. Correlations between DNA methylation status of EGRs and overall survival (OS) **(A)**, disease-free interval (DFI) **(B)**, or progression-free interval (PFI) **(C)**.

Information about promoter methylation of EGRs was obtained from the DNMIVD database. Differential DNA methylation status of EGRs between breast cancer and normal breast tissues were compared. As shown in Supplementary Figure S7A, EGRs were all differentially methylated genes (EGR1, *p* = 0.021; EGR2, *p* = 7.14e-3; EGR3, *p* = 3.94e-3; EGR4, *p* = 7.17e-5). Then we explored the correlations between methylation status and mRNA expression of EGRs in BRCA; other than EGR1 (Pearson r = 0.02, *p* = 6.25e-01; Spearman r = 0.02, *p* = 2.91e-1) and EGR4 (Pearson r = -0.04, *p* = 2.18e-01, Spearman r = −0.01, *p* = 8.44e-01), the DNA methylation status of other EGR family members showed weak correlations to their mRNA expression levels (EGR2, Pearson r = −0.13, *p* = 1.64e-04; Spearman r = −0.1, *p* = 3.62e-03; EGR3, Pearson r = −0.23, *p* = 6.75e-12; Spearman r = −0.29, *p* = 6.00e-18) (Supplementary Figure S7B,C). In subtype analysis, the DNA methylation of EGR1 was associated with ER status, PR status, and HER2 status; the DNA methylation of EGR2 was associated with PR; the DNA methylation of EGR3 was associated with ER status, PR status, and TNBC status; the DNA methylation of EGR4 was associated with ER status, HER2 status, and TNBC status (Supplementary Figure S8). In the following prognostic analysis, no significant results were found. The methylation status of all the EGR members showed no correlation to OS, disease-free interval (DFI), or progression-free interval (PFI) (Figure 4).

### Correlation and Functional Analyses of Early Growth Response Proteins

**FIGURE 5:**
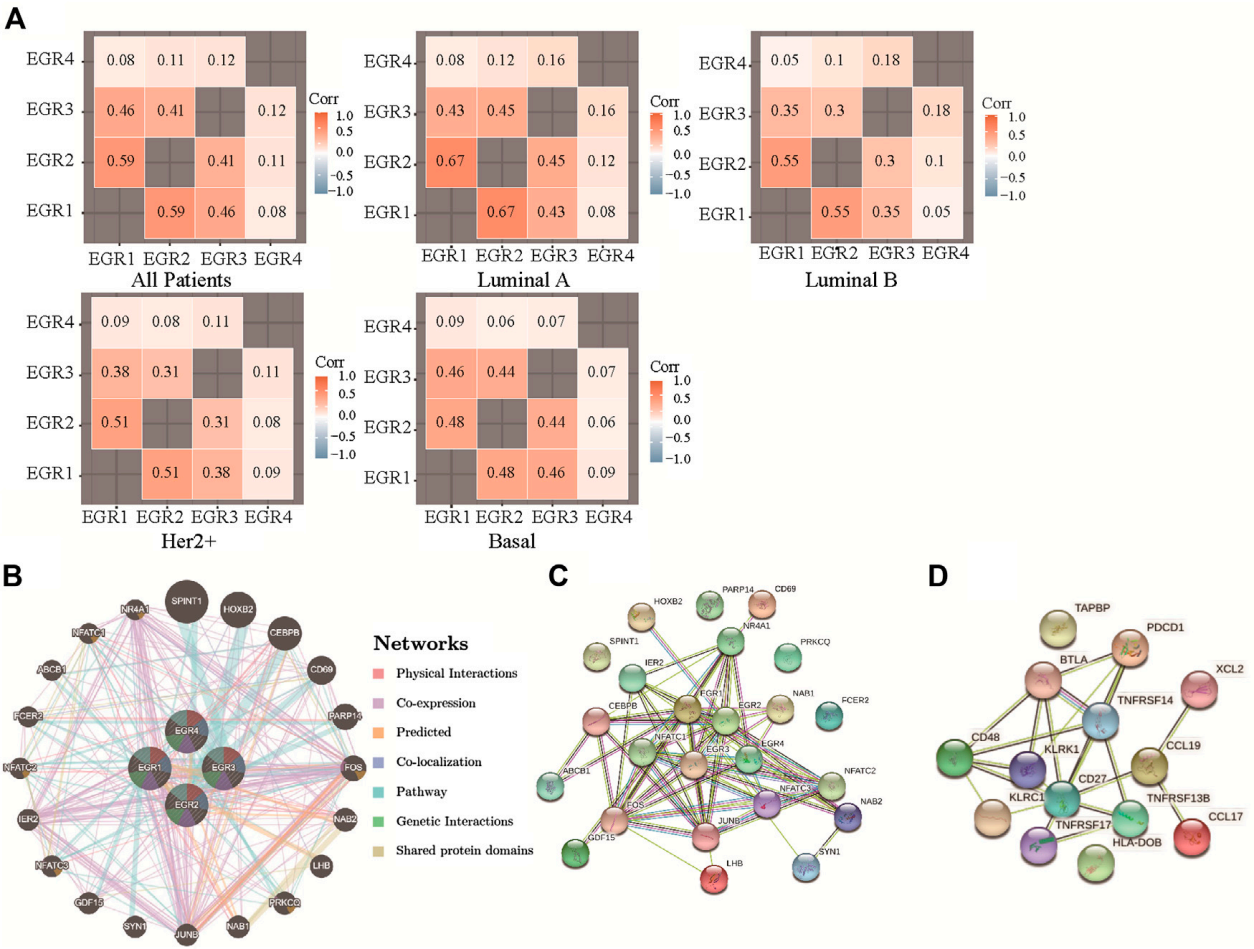
Correlations among expression levels of EGRs and interaction analyses in breast cancer. **(A)** Correlations among expression levels of EGRs by using all DNA microarray data, from bc-GenExMiner v4.5, heatmaps were replotted by R. **(B)** Gene-gene interaction network among EGRs in the GeneMANIA dataset. **(C)** Protein-protein interaction network among EGRs in the STRING dataset. **(D)** Protein-protein interaction network among 14 signature genes in the STRING dataset.

By using the bc-GenExMiner v4.5, we acquired the information of correlations among EGRs in all patients and different subtypes and replotted the heatmaps by using R. With data from all DNA microarray, we found the strongest positive correlation between EGR1 and EGR2 and the weakest positive correlation between EGR4 and other EGRs in all patients; the same pattern was detected in all subtypes (Figure 5A). We verified the coexpression pattern of EGRs by using data from all RNA-seq (Supplementary Figure S9). Then we analyzed the relationship of EGRs at the gene level by using the GeneMANIA database (Figure 5B). No physical interactions were found among all EGRs. Coexpression was found among EGR1, EGR2, and EGR3, between EGR2 and EGR4. We identified interactions of EGRs at the protein expression level by using the STRING database (Figure 5C). EGR1 was shown to interact with EGR2 and EGR3 in coexpression and automated text mining; besides, relationships were noticed between EGR2 and EGR3 in coexpression, database annotated, and automated text mining. No correlations were found between EGR4 and other EGRs. We also obtained the top 20 genes (Supplementary Table S1) associated with EGRs from GeneMANIA; together with EGRs, we performed GO and KEGG pathway analyses via the STRING database. Detailed results were shown in Table 2. The top 5 terms in the BP category were transcription by RNA polymerase II, positive regulation of macromolecule biosynthetic process, positive regulation of transcription by RNA polymerase II, positive regulation of macromolecule metabolic process, and positive regulation of nitrogen compound metabolic process. The 5 most highly enriched functions in the CC category were transcription factor AP-1 complex, nucleus, nuclear chromosome, RNA polymerase II transcription factor complex, and transcription regulator complex. In the MF category, EGRs and their associated genes were mainly enriched in DNA-binding transcription activator activity (RNA polymerase II-specific), sequence-specific DNA binding, DNA-binding transcription factor activity (RNA polymerase II-specific), transcription regulator activity, and RNA polymerase II regulatory region sequence-specific DNA binding. Significant immune-related pathways were Th1 and Th2 cell differentiation, Th17 cell differentiation, T cell receptor signaling pathway, B cell receptor signaling pathway, IL-17 signaling pathway, and natural killer cell-mediated cytotoxicity.

**Table 2:** GO and KEGG pathway analyses of EGRs.

	Term description	False discovery rate
GO_BP	Transcription by RNA polymerase II	3.53E-08
	Positive regulation of macromolecule biosynthetic process	4.77E-08
	Positive regulation of transcription by RNA polymerase II	4.77E-08
	Positive regulation of macromolecule metabolic process	5.17E-08
	Positive regulation of nitrogen compound metabolic process	2.02E-07
GO_CC	Transcription factor AP-1 complex	0.0031
	Nucleus	0.0043
	Nuclear chromosome	0.0123
	RNA polymerase II transcription factor complex	0.0166
	Transcription regulator complex	0.0166
GO_MF	DNA-binding transcription activator activity, RNA polymerase II-specific	2.65E-12
	Sequence-specific DNA binding	2.31E-09
	DNA-binding transcription factor activity, RNA polymerase II-specific	3.92E-07
	Transcription regulator activity	4.83E-07
	RNA polymerase II regulatory region sequence-specific DNA binding	9.13E-07
KEGG	Th1 and Th2 cell differentiation	2.91E-06
	Th17 cell differentiation	3.40E-06
	T cell receptor signaling pathway	3.40E-06
	B cell receptor signaling pathway	2.25E-05
	IL-17 signaling pathway	0.0249
	Natural killer cell-mediated cytotoxicity	0.039

FDR < 0.05 was considered statistically significant. GO, Gene Ontology; KEGG, Kyoto Encyclopedia of Genes and Genomes; BP, biological processes; CC, cellular components; MF, molecular function; FDR, false discovery rate.

### Correlations Between Early Growth Response Proteins and Tumor-Infiltrating Immune Cells

**FIGURE 6:**
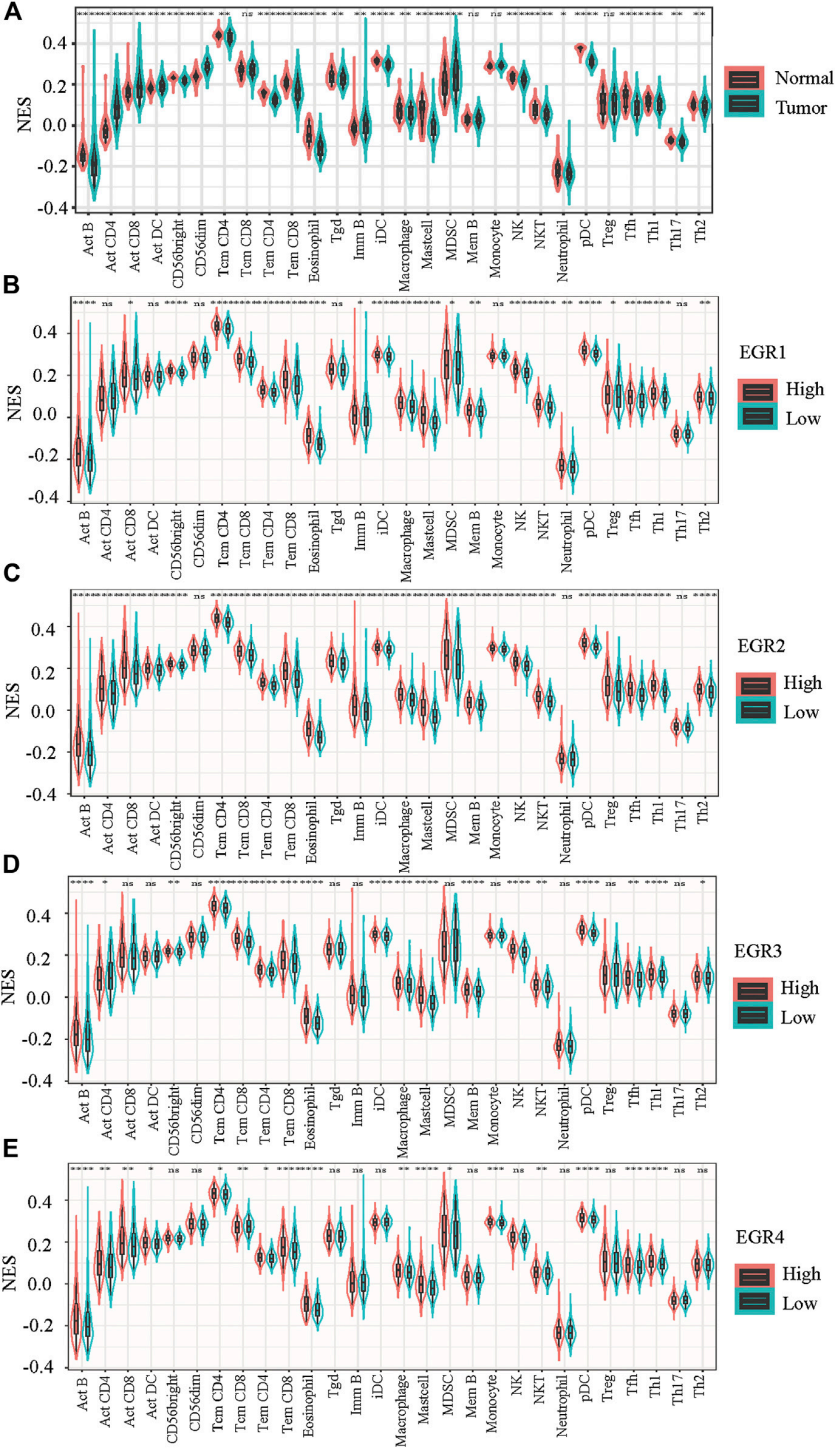
Evaluation of the proportions of 28 types of immune cell infiltration by ssGSEA in TCGA BRCA cohorts. **(A)** The differences in the immune cell distribution between malignant and normal in BRCA. **(B–E)** The immune cell distribution according to expression levels of EGRs in BRCA. Statistically significant differences were considered when *p* < 0.05 (****p < 0.0001, ***p < 0.001, **p < 0.01, and *p < 0.05).

**FIGURE 7:**
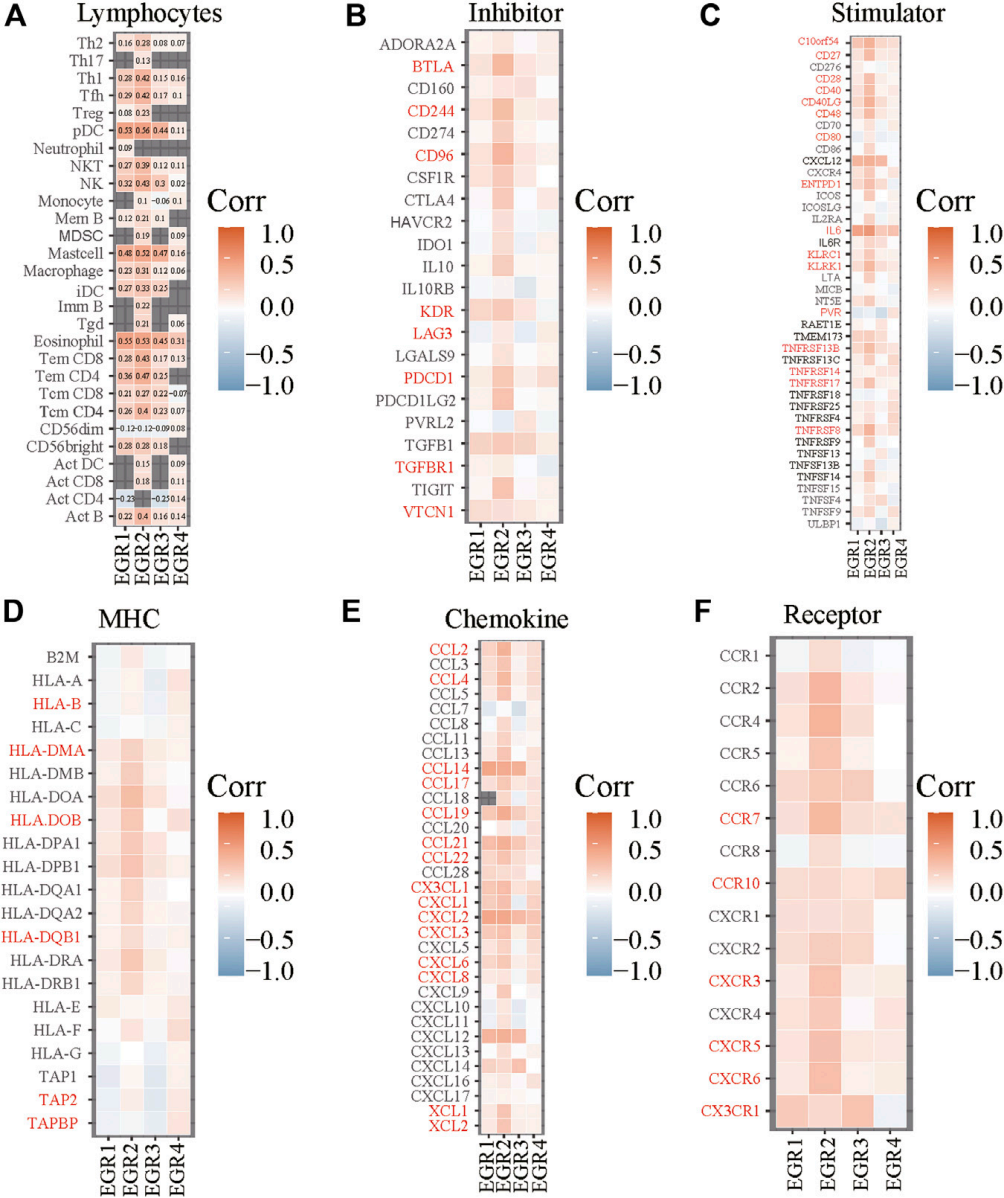
Correlations between EGRs and lymphocytes or immune-related genes. Correlations between expression levels of EGRs and lymphocytes **(A)**, immunoinhibitory **(B)**, immunostimulatory **(C)**, MHC **(D)**, chemokine **(E)**, or receptor **(F)**.

**FIGURE 8:**
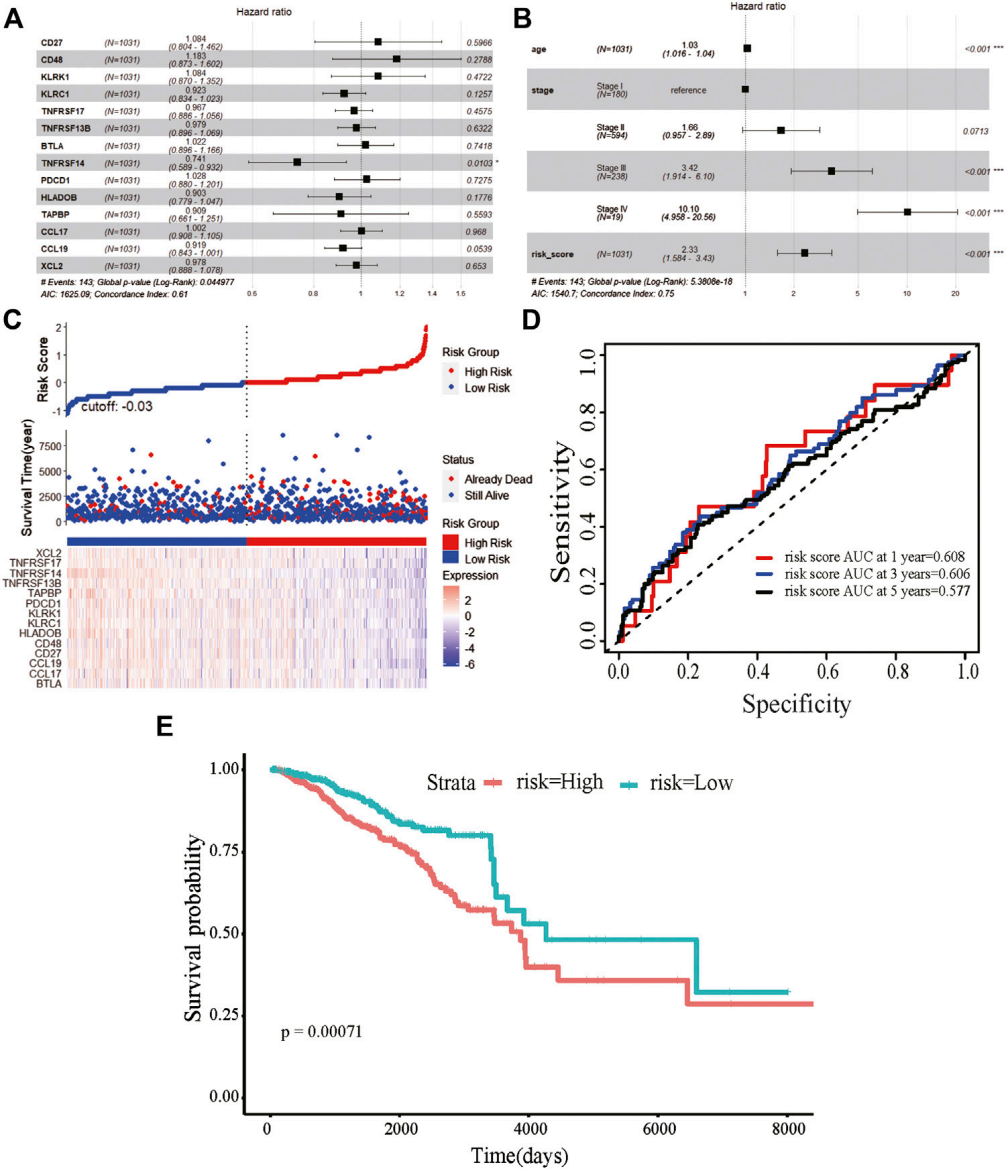
Prognostic value of the 14-gene signature based on EGRs-associated immune-related genes. **(A)** The hazard ratios of genes integrated into the prognostic signature by multivariate Cox regression analysis in breast cancer. **(B)** Multivariate Cox regression analysis of the risk score combined age and stage in breast cancer regarding overall survival (OS). **(C)** Distribution of risk score, along with survival statuses, and gene expression profiles for breast cancer. **(D)** Time-dependent receiver operating characteristic curves of the risk score at 1 year, 3 years, and 5 years for breast cancer. **(E)** Kaplan–Meier curve for breast cancer regarding the risk score (***p < 0.001, **p < 0.01, and *p < 0.05).

When compared with normal breast tissues, 24 out of 28 lymphocytes showed significant differential in BRCA, including Act B, Act CD4, Act CD8, Act DC, CD56 bright, CD56 dim, Tcm CD4, Tem CD4, Tem CD8, eosinophil, Tgd, Imm B, iDC, macrophage, mast cell, MDSC, NK, NKT, neutrophil, pDC, Tfh, Th1, Th2, and Th17 (Figure 6A). Then we attempted to find whether EGRs were associated with immune infiltration in BRCA. By using data from TCGA, patients with BRCA were divided into 2 groups (high expression and low expression), according to the expressions of EGRs, respectively. 22 out of 28 lymphocytes were affected by the expression of EGR1 (Figure 6B). Except CD56 dim, neutrophil, and Th17; the rest of the lymphocytes were all affected by EGR2 expression (Figure 6C). 18 out of 28 lymphocytes were affected by the expression of EGR3 expression (Figure 6D), and 17 lymphocytes were influenced by EGR4 expression (Figure 8E). Further correlation analysis demonstrated that 15 out of 28 lymphocytes were associated with all EGR members (*p* < 0.05) (Figure 7A).

### Construction of Risk Prediction Model

**FIGURE 9:**
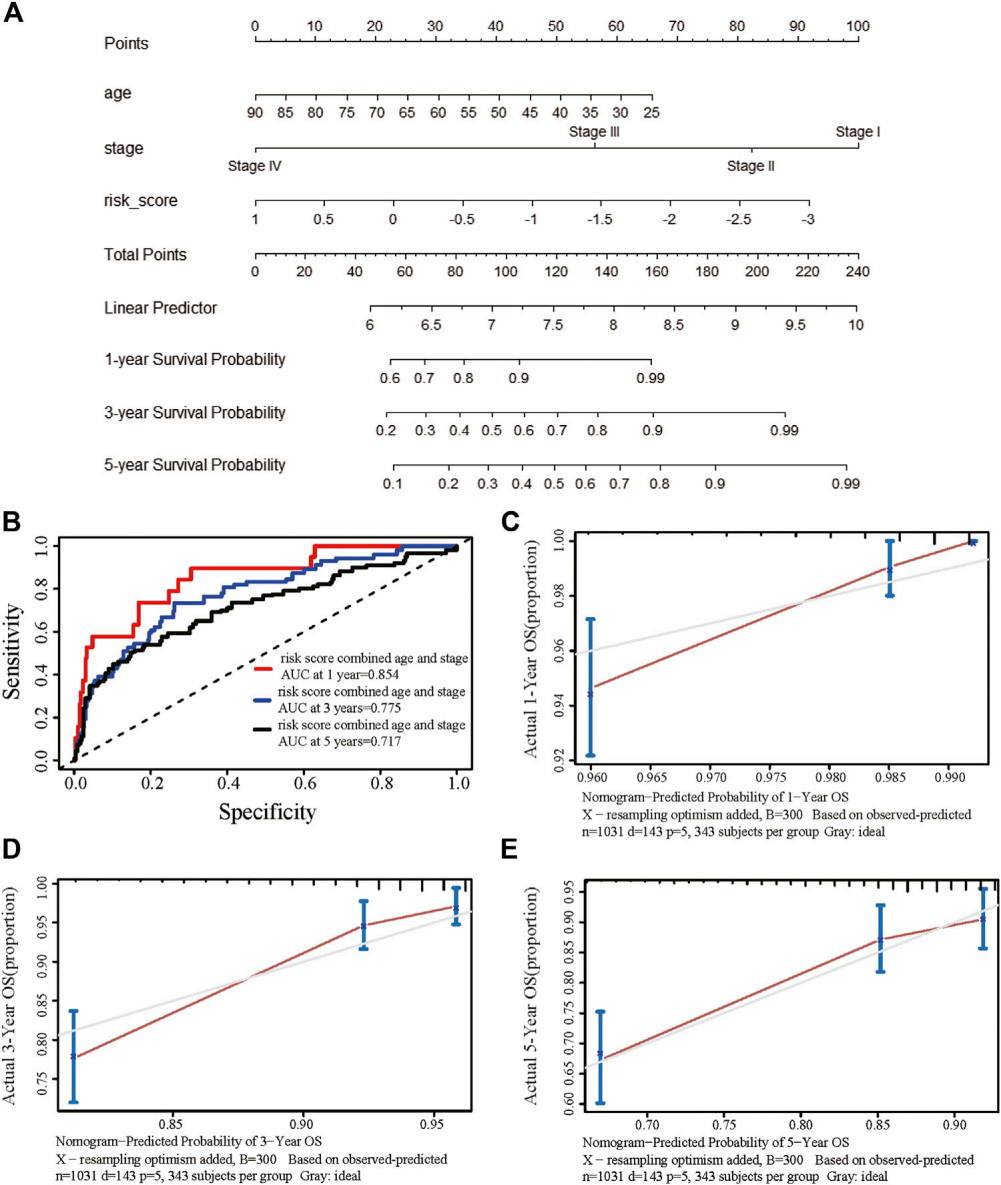
Establishment of the prognostic nomogram in breast cancer with the risk score combining age and stage. **(A)** A nomogram for predicting 1-, 3-, and 5-year survival possibilities of individual patients with breast cancer. **(B)** Time-dependent receiver operating characteristic curves of the risk score combined age and stage at 1 year, 3 years, and 5 years for breast cancer. The calibration curve of 1-year **(C)**, 3-year **(D)**, and 5-year **(E)** survival of breast cancer patients. The 45◦ dashed line represented a perfect uniformity between nomogram-predicted and real possibilities.

We obtained information on immune-related genes including immunoinhibitors, immunostimulators, MHCs, chemokines, and receptors via the TISIDB database. Detailed correlations between EGRs and the mentioned immune-related genes were available, and heatmaps were replotted by using R (Figures 7B–F), in which red font represented genes related to all EGRs. We entered these variables into the univariate Cox regression analysis, respectively, and acquired 14 genes that were associated with OS in BRCA, including CD27, CD48, KLRK1, KLRC1, TNFRSF17, TNFRSF13B, TNFRSF14, BTLA, PDCD1, HLADOB, TAPBP, CCL17, CCL19, and XCL2 (Supplementary Table S2). Detailed information on these genes is presented in Supplementary Table S3. Then the multivariate Cox regression analysis was performed to estimate the association between the 14-gene signature and OS (Figure 8A). The risk score was calculated by adding up the product of expression value and coefficient of each gene. As shown in Figures 8C–E, the expression levels of these 14 genes were higher in the low-risk group than in high-risk group; the area under the curve (AUC) values of the risk score were 0.608 at 1 year, 0.606 at 3 years, and 0.577 at 5 years; the Kaplan–Meier survival curve demonstrated that BRCA patients with low-risk scores achieved better survival as opposed to those with high-risk scores (*p* = 0.00071). Afterward, we entered the risk score, age, and stage into the multivariate Cox regression analysis. Accordingly, age, stage Ⅲ, stage Ⅳ, and the risk score acted as independent risk factors (Figure 8B). We also performed GO and KEGG pathway analyses on the 14 signature genes (Supplementary Table S4). The top 5 terms in the BP category were CCR chemokine receptor binding, immune system process, cellular response to tumor necrosis factor, T cell costimulation, and positive regulation of T cell activation. The 5 most highly enriched functions in the CC category were intrinsic component of membrane, side of membrane, external side of plasma membrane, intrinsic component of plasma membrane, and plasma membrane. In the MF category, the signature genes were mainly enriched in CCR chemokine receptor binding, chemokine activity, signaling receptor activity, MHC protein complex binding, and MHC class I protein binding. Lastly, a prognostic nomogram in BRCA was constructed to anticipate the individuals’ survival probability by weighing risk score, age, and stage (Figure 9A). The concordance index (C-index) was 0.75. The AUC values were 0.854 at 1 year, 0.775 at 3 years, and 0.717 at 5 years (Figure 9B). Calibration was conducted for the nomogram. It showed that the nomogram-predicted probability (solid line) well matched the idea reference line (dash line) for the 5-year survival (Figures 9C–E). We verified the risk prediction model by using the METABRIC cohort, however, in which the data of XCL2 was unavailable and the validation was performed with a 13-gene signature. The AUC values of risk score combining age and stage were 0.888 at 1 year, 0.768 at 3 years, and 0.726 at 5 years (Supplementary Figure S10B).

## Discussion

Breast cancer, as a systemic disease, should be treated by a multimodality approach. Local and systemic therapies are both play very important roles in the treatment of breast cancer. Even though breast cancers have a relatively good prognosis, such as patients with positive hormone receptor, recurrences may be found many years after treatment. Though the 5-year survival rate for breast cancer has been significantly improved, over 90% (Tokumaru et al., 2020), it is not completely understood why recurrences emerge after over a decade. One of the theories suggests that the immune system sequesters isolated cancer cells; when some event disturbs the immunologic equipoise, it cannot suppress the growth of the tumor (Tokumaru et al., 2020). Numerous efforts have been done to convert breast cancer, which is regarded as an immunologically “cold” tumor, to an immunologically “hot” disease. However, the success of immunotherapeutic drugs such as immune checkpoint inhibitors (ICIs) in the treatment of melanoma and lung cancer has not been turned into a reality in breast cancer.

As previously reported, EGR1 plays multiple tumor suppressor roles in breast cancer, in which its expression is often lost (Crawford et al., 2019); EGR2 is a growth inhibitor when upregulated in tumor cells (Salotti et al., 2015), exogenous expression of EGR2 can also suppress the growth of tumor cells (Unoki and Nakamura, 2003), but its role in breast cancer is rarely reported; downregulation of EGR3 can promote the migration and invasion of hepatocellular carcinoma (HCC) (Wangdong et al., 2017), high expression level of EGR3 was discovered in prostate cancer samples (Pio et al., 2013), but its role in breast cancer is also rarely reported. Few researches reported EGR4 in malignant tumors; only some articles reported that EGR4 might promote NSCLC (He et al., 2019), small cell lung cancer (SCLC) (Matsuo et al., 2014), and cholangiocarcinoma (CHOL) to develop (Gong et al., 2020). In this study, we first analyzed the expressions of EGRs and their correlations with the clinicopathological characteristics in breast cancer. The EGR family members, except EGR4, were all genes with differential expressions in breast cancer. In all subtypes of breast cancer, the mRNA levels of EGR1, EGR2, and EGR3 were all lower in malignant breast tissues compared with normal tissues. Moreover, we found that the expression of EGR1, EGR2, and EGR3 decreased as the tumors progressed. The expression levels of these 3 genes were found to be the lowest in SBR3, NPI3, and stage Ⅳ subgroups and the highest in less aggressive subgroups, including SBR1, NPI1, and stage Ⅰ, demonstrating that EGR1, EGR2, and EGR3 might work as tumor suppressor genes. EGR4 can be regarded as a nonexpressing gene in breast cancer, suggesting that it might be neither promoter nor suppressor of breast cancer.

Then we investigated the prognosis significance of the EGR family in breast cancer with all DNA microarray data and verified it by using all RNA-seq data via bc-GenExMiner v4.5 database. The results demonstrated that higher levels of EGRs were associated with better OS and DFS in breast cancer, except EGR4. In subtype analysis, EGR2 was associated with OS and DFS in Luminal A and basal subtype and DFS in Her2+ subtype; EGR3 was associated with OS and DFS in Luminal A subtype; interestingly, EGR1 showed no prognostic value in all subtypes. As to the prognosis significance of the EGR family, no clinical trials have been reported so far. For the prognostic value of the EGR family in breast cancer, similar conclusions were drawn by Yuchang Fei and colleagues by using public databases (Fei et al., 2019), but they had not further explored the potential mechanism behind that. Hence, we attempted to explore the potential mechanism capable of explaining why higher expression levels of EGR1, EGR2, and EGR3 predicted better outcomes in patients with breast cancer. Then we first focused on the DNA level. In genetic alteration analysis, we discovered a moderate correlation between EGR3 mRNA expression level and genetic alteration, weak correlations between mRNA levels of other EGR family members, and genetic alterations. However, genetic alterations of EGRs were not associated with OS of DFS. In DNA methylation analysis, though the DNA methylation status of all EGRs was higher in breast cancer than in normal tissues, they showed no correlation with the outcome either. So we concluded that neither genetic alteration nor DNA methylation contributed to the prognosis significance of EGRs in breast cancer. Afterward, we acquired the top 20 genes associated with EGRs from GeneMANIA; together with EGRs, functional analysis was performed in the STRING database. Several immune-related pathways were found in the KEGG pathway analysis, in which Th1, Th2, Th17, T cell receptor, B cell receptor, IL-17, and natural killer cell were involved. The correlation between EGRs and the immune system was previously proved. EGR1 is of high importance for thymocyte development, positive selection of CD4 and CD8 single-positive cells, and macrophage lineage differentiation (Krishnaraju et al., 1995; Bettini et al., 2002). EGR2 and EGR3 are noticeably related, critically control the self-tolerance of lymphocytes and the development of NKT cells, and are induced in both naive and tolerant T cells (Harris et al., 2004; Safford et al., 2005; Anderson et al., 2009). Th1 differentiation and anticancer immunity are reported to be regulated by EGR4 in vivo (Mookerjee-Basu et al., 2020).

Public databases with high-throughput gene expression datasets are available nowadays and facilitate the discovery of potential markers that are more reliable and robust in various cancers. Ascierto et al. reported an immune-related gene signature associated with recurrence-free survival in breast cancer patients with microarray data (Ascierto et al., 2012). Jianqun Ma et al. built a risk prediction model with a 14-gene prognostic signature in LUAD and a 13-gene prognostic signature in lung squamous cell carcinoma (LUSC) with data retrieved from public databases. Subsequently, they established a nomogram in LUAD to predict the individuals’ survival probability by using risk score combined with clinical features; the accuracy (C-index) reached 0.71 (Zhu et al., 2020). Consistently, with immune-related genes associated with EGRs, we established an immune gene signature for breast cancer. The risk score derived from the gene signature displayed a noticeable relationship to OS in breast cancer. But the C-index only reached 0.61; AUC values only reached 0.608 at 1 year, 0.606 at 3 years, and 0.577 at 5 years. However, the prognosis accuracy was more significantly elevated by the composition of age and stage, with C-index reaching 0.75 and AUC values reaching 0.854 at 1 year, 0.775 at 3 years, and 0.717 at 5 years in TCGA breast cancer cohort. Our results demonstrated that the risk score derived from EGRs-associated immune genes was able to discriminate risk groups defined by a set of signature genes, which may facilitate the development of the well-verified signature for cancer prognoses and treatments.

Several limitations should be addressed here. All the analyses were carried out by exploiting public datasets, requiring the validation in the in-house patients. The correlation between EGRs and the immune system should be further explored. Furthermore, the prognosis significances of EGRs and the predictive ability of the risk model should be verified via clinical trials. Lastly, it is also important to understand the regulation network of EGRs, which work as transcript factors, and the mechanism of the 14-gene signature.

In conclusion, the results here suggested that, except EGR4, higher levels of other EGR family members indicated better OS and DFS in breast cancer. DNA methylation status and genetic alterations did not contribute to the prognostic significance. EGR family, as a whole, might be critical to tumor immune microenvironments. The risk prediction model based on 14 immune genes associated with EGRs could predict the overall survival rate for patients with breast cancer. Prospective studies should be conducted for verifying biomarker’s clinically related uses in personalized breast cancer management.

## Data Availability Statement

The datasets presented in this study can be found in online repositories. The names of the repository/repositories and accession numbers can be found in the article/Supplementary Material.

## Author Contributions

XZ designed and performed the study, analyzed and interpreted data, and drafted the manuscript. FZ and YL participated in data analysis and figure preparation. DW revised the manuscript critically. All authors read and approved the final manuscript.

## Funding

This study was supported by funding from Zibo Maternal and Child Care Hospital.

## Conflict of Interest

The authors declare that the research was conducted in the absence of any commercial or financial relationships that could be construed as a potential conflict of interest.
