# Supplementary material for: A Risk Prediction Model for Breast Cancer Based on Immune Genes Related to Early Growth Response Proteins Family
Source: Front Mol Biosci. 2021 Feb 3;7:616547. doi: 10.3389/fmolb.2020.616547 (PMC7887293; doi:10.3389/fmolb.2020.616547)
Supplement: Supplementary file 1 [file datasheet1.pdf]

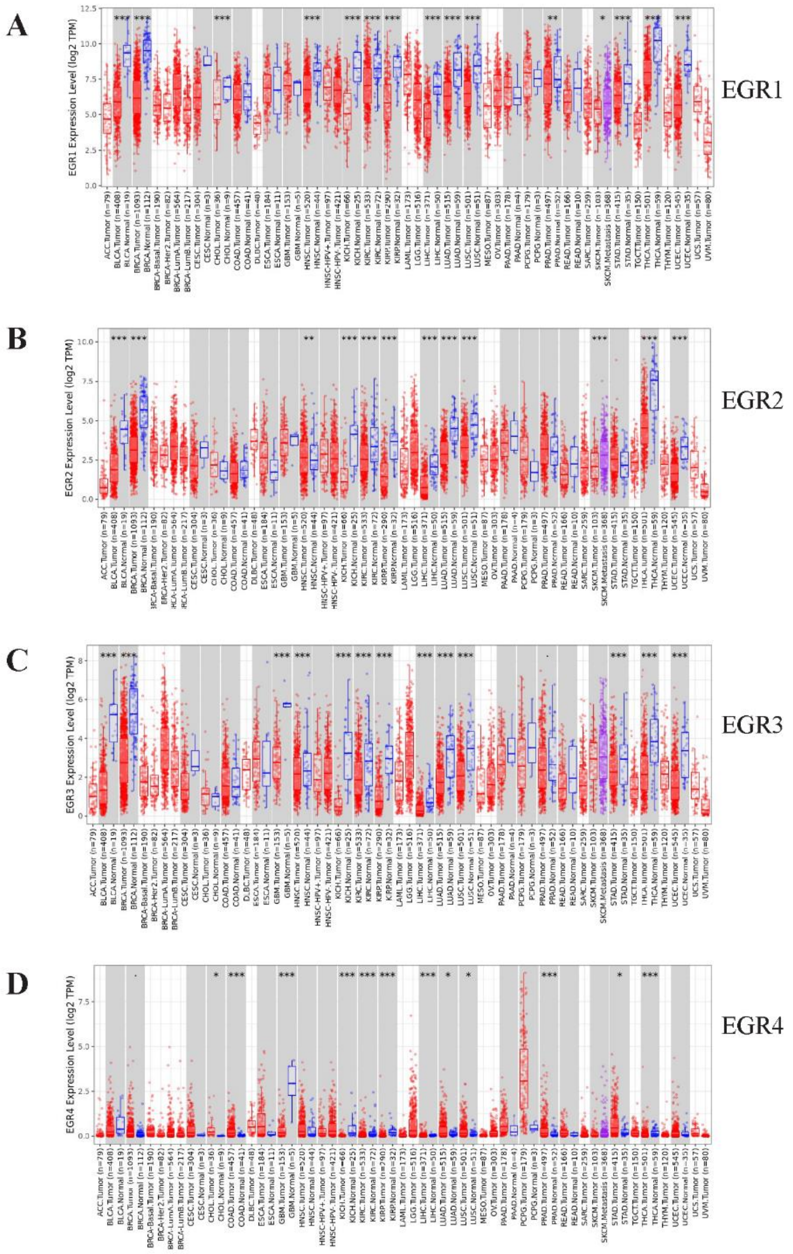

Supplementary Figure 1 Expression levels of EGRs in pan-cancer.
(A) Expression of EGR1 in pan-cancer. (B) Expression of EGR2 in pan-cancer. (C) Expression of EGR3 in pan-cancer. (D) Expression of EGR4 in pan-cancer. (***P < 0.001, **P < 0.01, *P < 0.05).

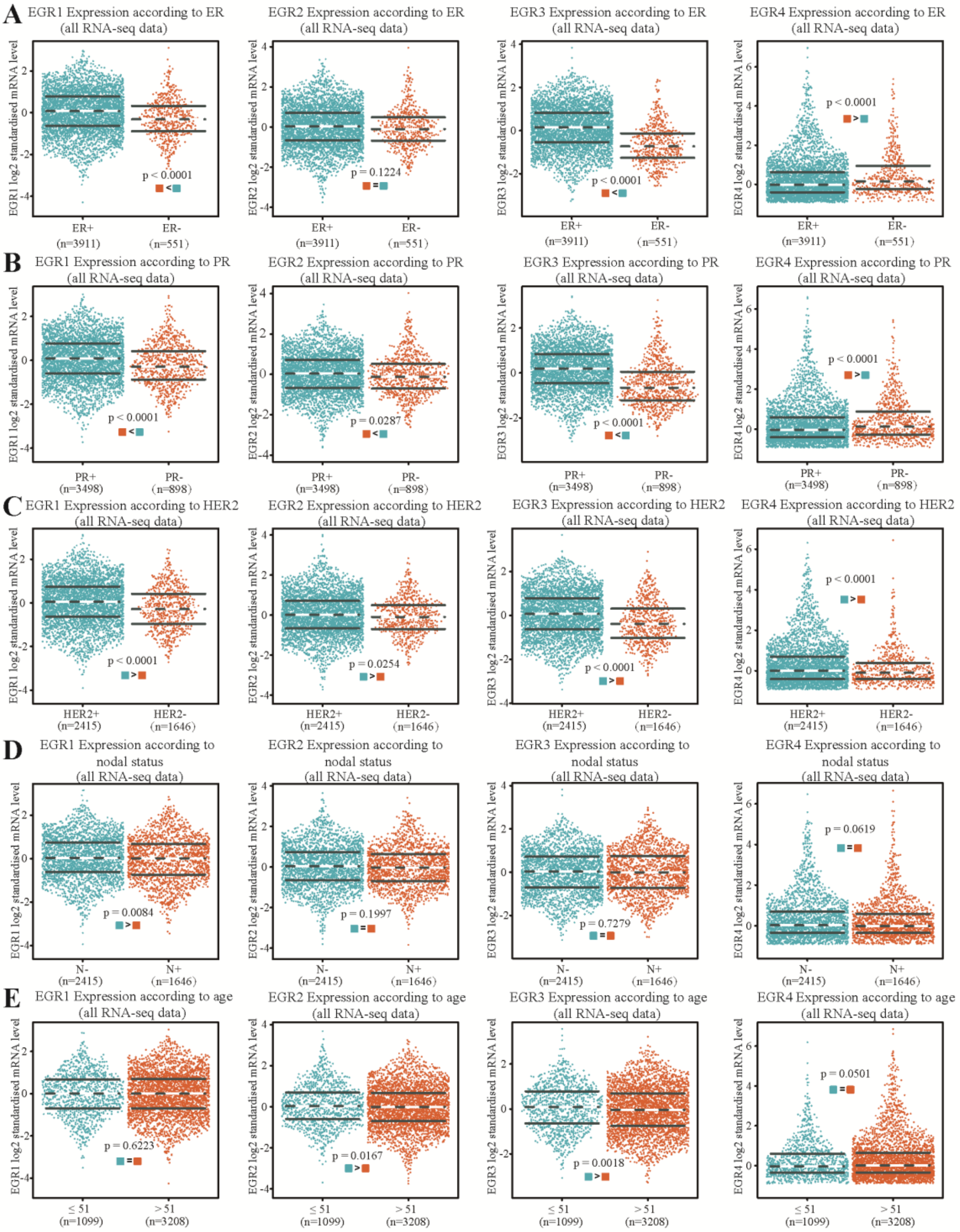

Supplementary Figure 2 Correlations between the expression levels of EGRs and clinical indicators in breast cancer.

(A) Correlations between the expression levels of EGRs and ER status. (B) Correlations between the expression levels of EGRs and PR status (C) Correlations between the expression levels of EGRs and HER2 status. (D) Correlations between the expression levels of EGRs and nodal status. (E) Correlations between the expression levels of EGRs and age.

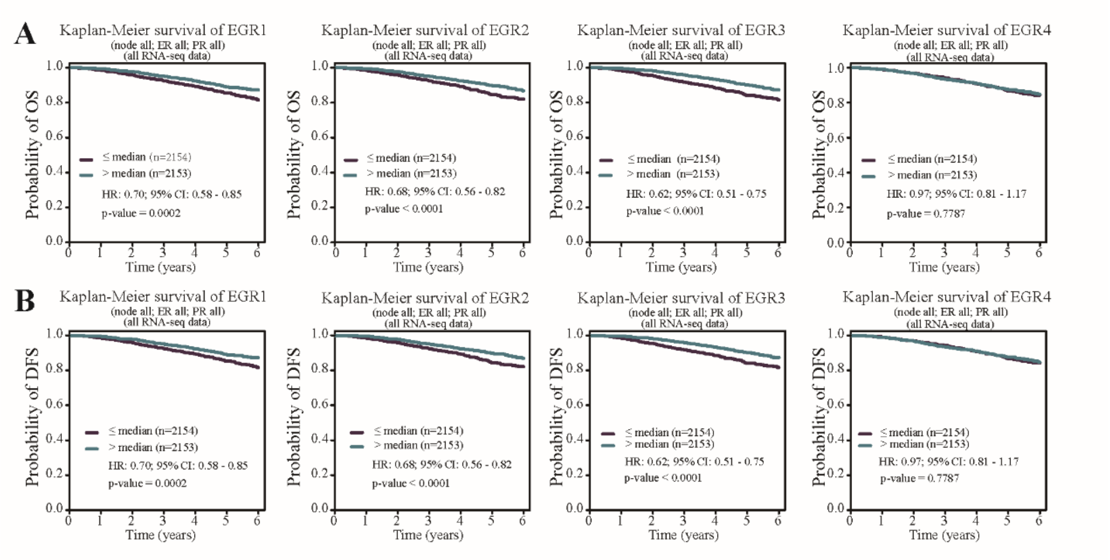

Supplementary Figure 3 Prognostic Value of EGR family in breast cancer (all RNA-seq data from bc-GenExMiner v4.5).

(A) Except EGR4, other members of EGR family were associated with OS, higher levels indicating better outcome (EGR1, p=0.0002; EGR2, p<0.0001; EGR3, p<0.0001; EGR4, p=0.7787). (B) Except EGR4, other members of EGR family were associated with DFS, higher levels indicating better outcome (EGR1, p=0.0002; EGR2, p<0.0001; EGR3, p<0.0001; EGR4, p=0.7787).

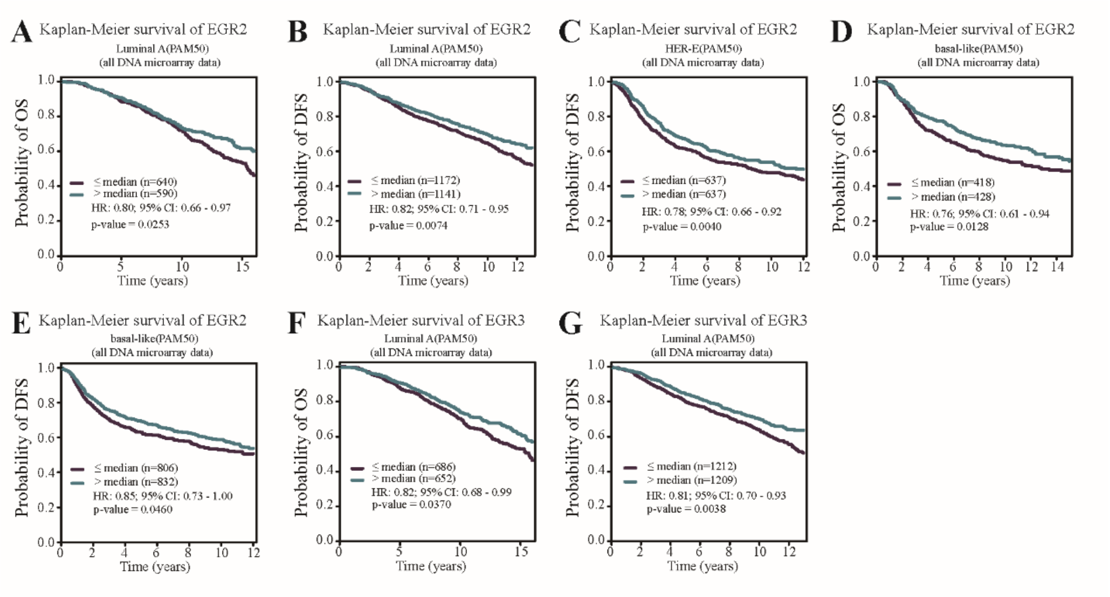

Supplementary Figure 4 Prognostic significance of EGR family in subtypes of breast cancer (all DNA microarray data from bc-GenExMiner v4.5).

The expression of EGR2 was associated with OS (p=0.0253) (A)and DFS (p=0.0074) (B) in Luminal A subtype. The expression of EGR2 was associated with DFS (p=0.0253) (C) in HER2-E subtype. The expression of EGR2 was associated with OS (p=0.0128) (D)and DFS (p=0.0460) (E) in Luminal A subtype. The expression of EGR3 was associated with OS (p=0.0370) (A)and DFS (p=0.0038) (B) in Luminal A subtype.

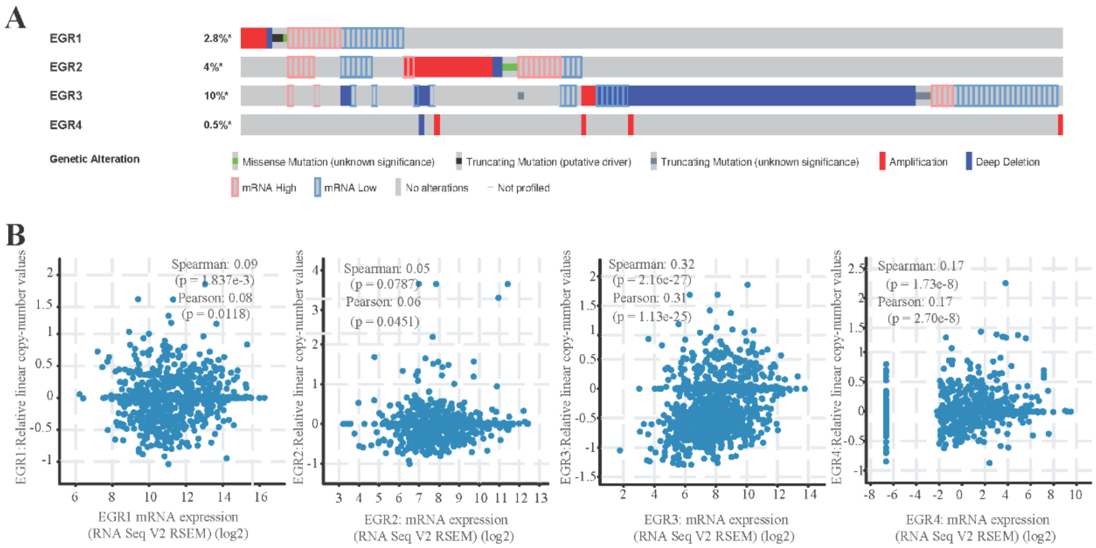

Supplementary Figure 5 Gene alterations of EGRs in breast invasive carcinoma. (A) Various genetic alterations in the EGR family sample. Databases: Breast Invasive Carcinoma (TCGA, Firehose Legacy). (B) Associations between copy number alterations of EGRs and their mRNA expression levels respectively.

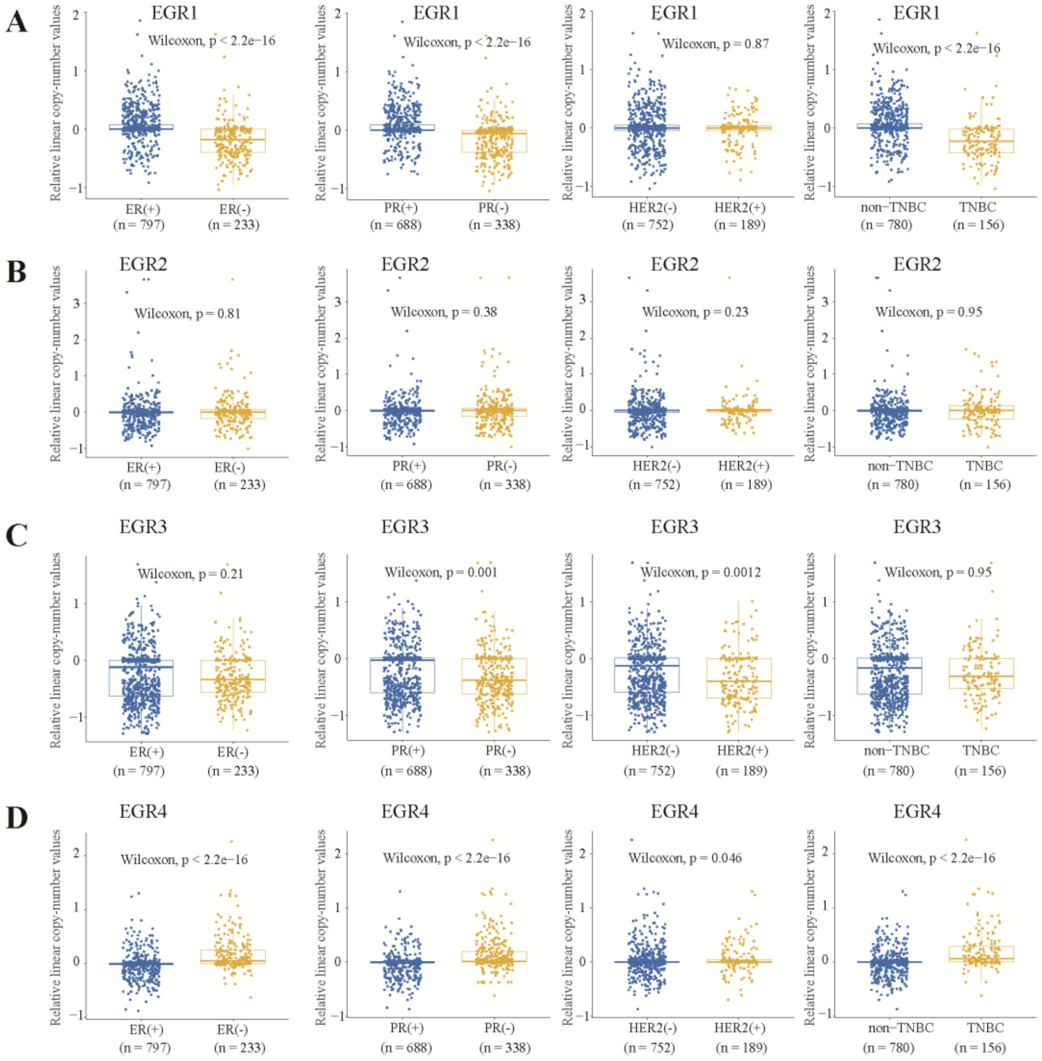

Supplementary Figure 6 Subtype analysis of EGRs genetic alteration in breast invasive carcinoma.

Relative linear copy-number values of EGR1 (A), EGR2 (B), EGR3 (C), and EGR4 (D) according to ER status, PR status, HER2 status, and TNBC status.

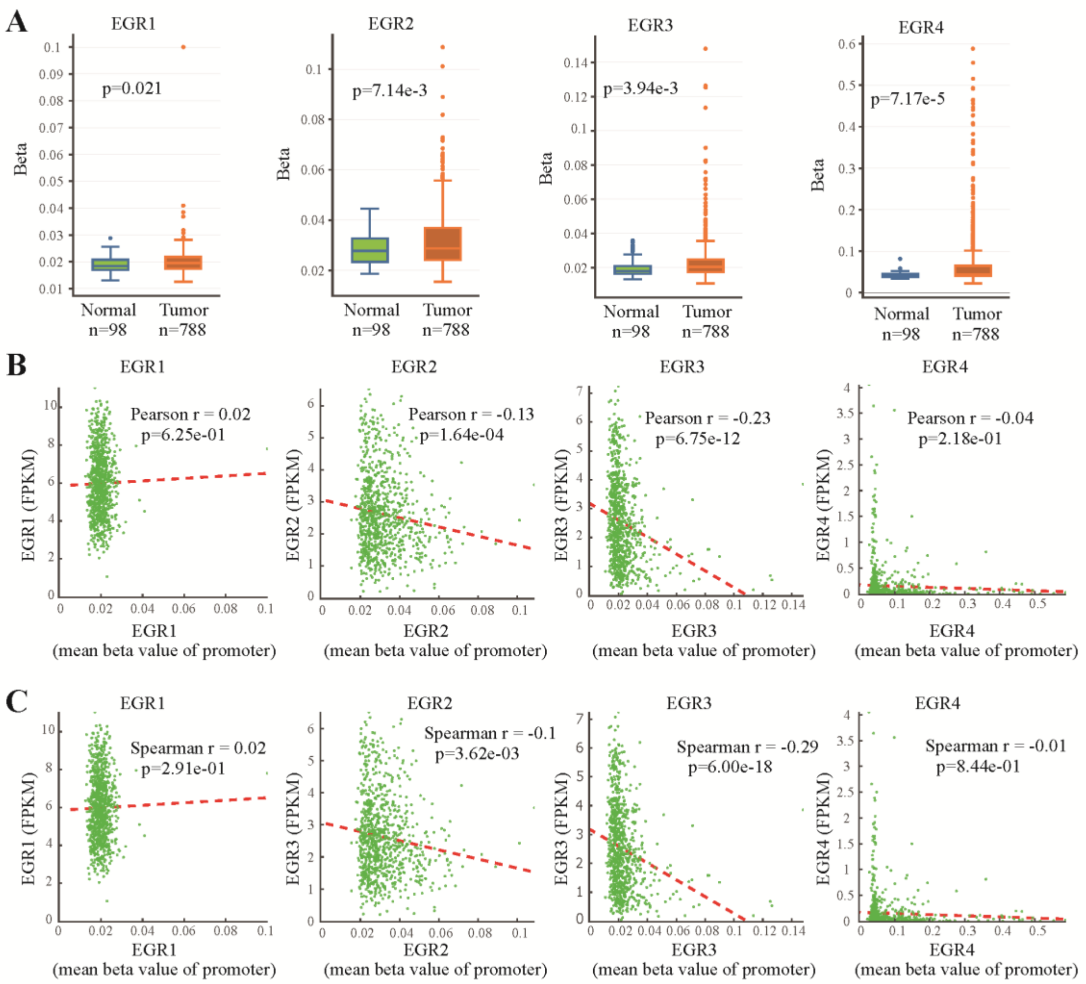

Supplementary Figure 7 DNA methylation status of EGRs in breast cancer.
(A) DNA methylation status of EGRs in breast cancer and normal breast tissues. (B) Correlations between DNA methylation status of EGRs and their mRNA expression levels respectively (Pearson correlation coefficient). (C) Correlations between DNA methylation status of EGRs and their mRNA expression levels respectively (Spearman’s correlation coefficient).

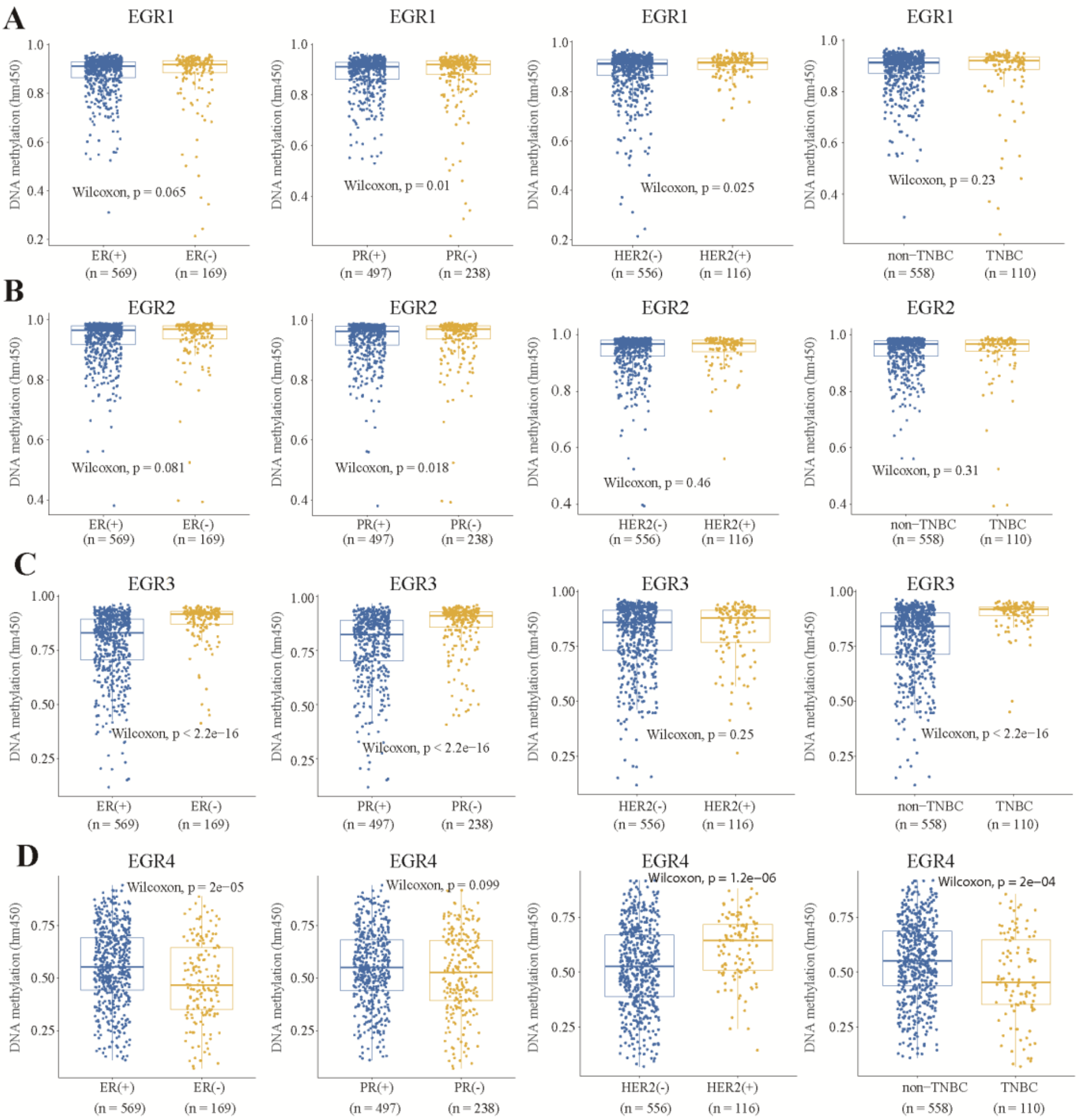

Supplementary Figure 8 Subtype analysis of EGRs DNA methylation in breast invasive carcinoma.

DNA methylation of EGR1 (A), EGR2 (B), EGR3 (C), and EGR4 (D) according to ER status, PR status, HER2 status, and TNBC status.

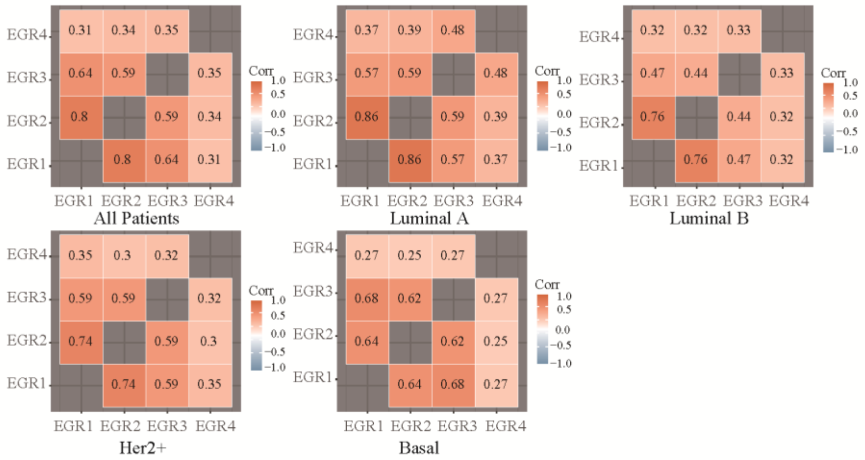

Supplementary Figure 9 Correlations among expression levels of EGRs

Correlations among expression levels of EGRs by using all RNA-seq data from bc-GenExMiner v4.5, heatmaps were replotted by R.

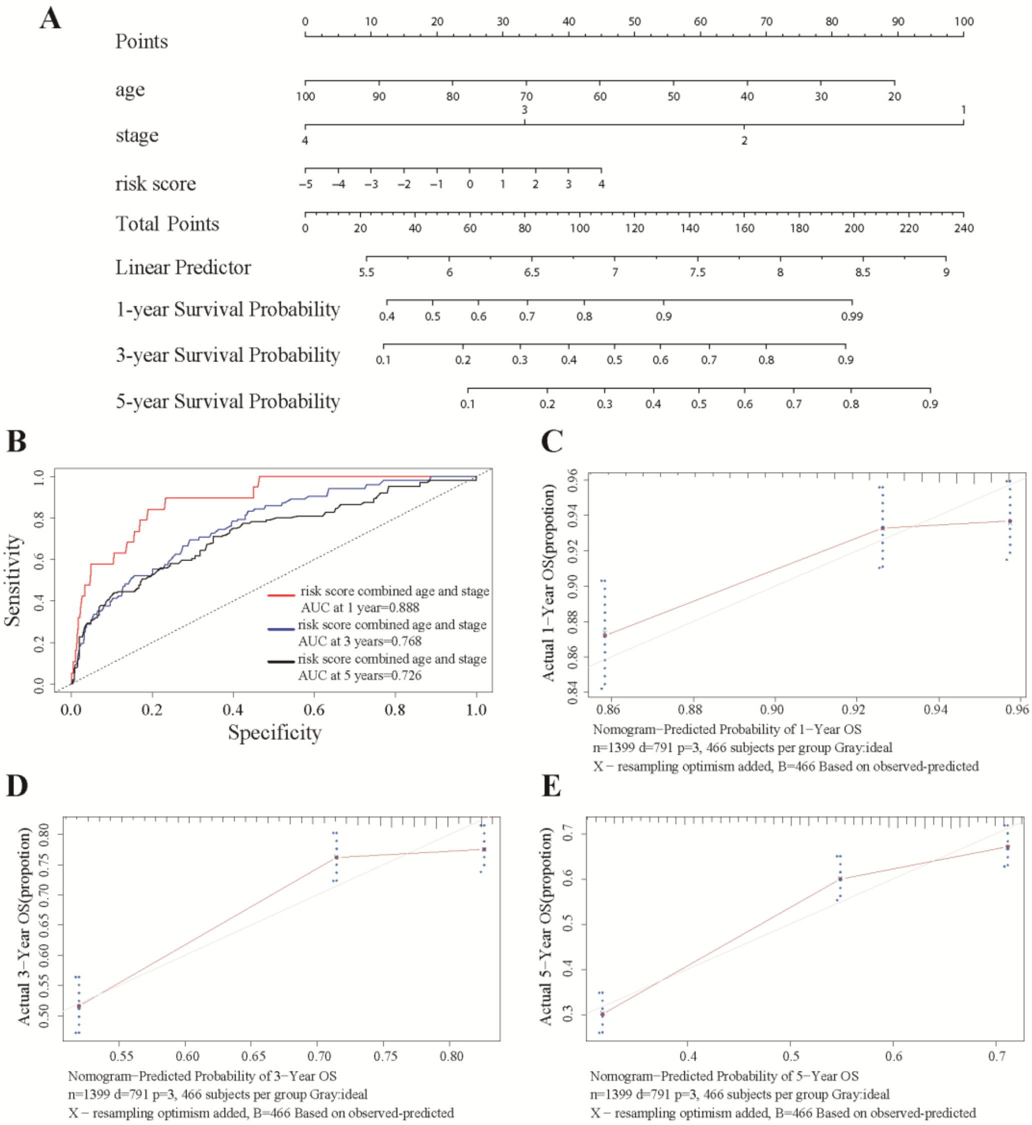

Supplementary Figure 10 Validation of risk prediction model with Metabric cohort

(A) A nomogram for predicting 1-, 3- and 5-year survival possibilities of individual patients with breast cancer. (B) Time-dependent receiver operating characteristic curves of the risk score combined age and stage at 1 year, 3 years, and 5 years for breast cancer. The calibration curve of 1-year (C), 3-year (D), and 5-year (E) survival of breast cancer patients. The 45◦ dashed line represented a perfect uniformity between nomogram-predicted and real possibilities.
