# Supplementary material for: A Risk Prediction Model for Breast Cancer Based on Immune Genes Related to Early Growth Response Proteins Family
Source: Front Mol Biosci. 2021 Feb 3;7:616547. doi: 10.3389/fmolb.2020.616547 (PMC7887293; doi:10.3389/fmolb.2020.616547)
Supplement: Supplementary file 2 [file datasheet2.pdf]

Supplementary Table 1 Top 20 genes related to EGRs according to GeneMANIA.

Gene	Description	Rank
SPINT1	serine peptidase inhibitor, Kunitz type 1 [Source: HGNC Symbol; Acc: HGNC:11246]	1
HOXB2	homeobox B2 [Source: HGNC Symbol; Acc: HGNC:5113]	2
CEBPB	CCAAT/enhancer binding protein beta [Source: HGNC Symbol; Acc: HGNC: 1834]	3
CD69	CD69 molecule [Source: HGNC Symbol; Acc: HGNC:1694]	4
PARP14	Poly (ADP-ribose) polymerase family member 14 [Source: HGNC Symbol; Acc: HGNC:29232]	5
FOS	Fos proto-oncogene, AP-1 transcription factor subunit [Source: HGNC Symbol; Acc: HGNC:3796]	6
NAB2	NGFI-A binding protein 2 [Source: HGNC Symbol; Acc: HGNC:7627]	7
LHB	luteinizing hormone beta polypeptide [Source: HGNC Symbol; Acc: HGNC: 6584]	8
PRKCQ	protein kinase C theta [Source: HGNC Symbol; Acc: HGNC:9410]	9
NAB1	NGFI-A binding protein 1 [Source: HGNC Symbol; Acc: HGNC:7626]	10
JUNB	JunB proto-oncogene, AP-1 transcription factor subunit [Source: HGNC Symbol; Acc: HGNC:6205]	11
SYN1	synapsin I [Source: HGNC Symbol; Acc: HGNC:11494]	12
GDF15	growth differentiation factor 15 [Source: HGNC Symbol; Acc: HGNC:30142]	13
NFATC3	nuclear factor of activated T-cells 3 [Source: HGNC Symbol; Acc: HGNC: 7777]	14
IER2	immediate early response 2 [Source: HGNC Symbol; Acc: HGNC:28871]	15
NFATC2	nuclear factor of activated T-cells 2 [Source: HGNC Symbol; Acc: HGNC: 7776]	16
FCER2	Fc fragment of IgE receptor II [Source: HGNC Symbol; Acc: HGNC:3612]	17
ABCB1	ATP binding cassette subfamily B member 1 [Source: HGNC Symbol; Acc: HGNC:40]	18
NFATC1	nuclear factor of activated T-cells 1 [Source: HGNC Symbol; Acc: HGNC: 7775]	19
NR4A1	nuclear receptor subfamily 4 group A member 1 [Source: HGNC Symbol; Acc: HGNC:7980]	20

Supplementary Table 2 Immune genes associated with OS in BRCA according to unilabiate cox regression.

Gene symbol	Type	Beta	HR (95% CI)	P value
CD27	immunostimolator	-0.10589	0.8995(0.8272-0.9781)	0.0132
CD48	immunostimolator	-0.11303	0.8931(0.7989-0.9985)	0.0469
KLRK1	immunostimolator	-0.10377	0.9014(0.8239-0.9862)	0.0237
KLRC1	immunostimolator	-0.08199	0.9213(0.8636_0.9828)	0.0129
TNFRSF17	immunostimolator	-0.06115	0.9407(0.8976-0.9858)	0.0105
TNFRSF13B	immunostimolator	-0.06385	0.9382(0.8954-0.9829)	0.0073
TNFRSF14	immunostimolator	-0.30505	0.7371(0.6166-0.8811)	8.00E-04
BTLA	immunoinhibitor	-0.07033	0.9321(0.8706-0.9979)	0.0434
PDCD1	immunoinhibitor	-0.08041	0.9227(0.856-0.9947)	0.0359
HLADOB	MHC	-0.12337	0.8839(0.807-0.9682)	0.0079
TAPBP	MHC	-0.3039	0.738(0.5581-0.9758)	0.033
CCL17	chemokine	-0.08632	0.9173(0.8543-0.985)	0.0175
CCL19	chemokine	-0.07588	0.9269(0.8863-0.9694)	9.00E-04
XCL2	chemokine	-0.06909	0.93324(0.881-0.9886)	0.0188

P<0.05 was considered statistically significant.

Supplementary Table 3 Functions of the genes included in the prognostic signature.

Gene symbol	Name	Type	Function
CD27	CD27 molecule	Immunostimulator	Receptor for CD70/CD27L. May play a role in survival of activated T-cells. May play a role in apoptosis through association with SIVA1.
CD48	CD48 molecule	Immunostimulator	Ligand for CD2. Might facilitate interaction between activated lymphocytes. Probably involved in regulating T-cell activation.
KLRK1	Killer cell lectin-like receptor subfamily K, member 1	Immunostimulator	Function as an activating and costimulatory receptor involved in immunosurveillance upon binding to various cellular stress-inducible ligands displayed at the surface of autologous tumor cells and virus-infected cells.
KLRC1	Killer cell lectin-like receptor subfamily C, member 1	Immunostimulator	Plays a role as a receptor for the recognition of MHC class I HLA-E molecules by NK cells and some cytotoxic T-cells.
TNFRSF17	Tumor necrosis factor receptor superfamily, member 17	Immunostimulator	Receptor for TNFSF13B/BLyS/BAFF and TNFSF13/APRIL. Promotes B-cell survival and plays a role in the regulation of humoral immunity. Activates NF-kappa-B and JNK.
TNFRSF13B	Tumor necrosis factor receptor superfamily, member 13B	Immunostimulator	Receptor for TNFSF13/APRIL and TNFSF13B/TALL1/BAFF/BLYS that binds both ligands with similar high affinity. Mediates calcineurin-dependent activation of NF-AT, as well as activation of NF-kappa-B and AP-1. Involved in the stimulation of B- and T-cell function and the regulation of humoral immunity.
TNFRSF14	Tumor necrosis factor receptor superfamily, member 14	Immunostimulator	Receptor for BTLA. Receptor for TNFSF14/LIGHT and homotrimeric TNFSF1/lymphotoxin-alpha. Involved in lymphocyte activation. Plays an important role in HSV pathogenesis because it enhanced the entry of several wild-type HSV strains of both serotypes into CHO cells, and mediated HSV entry into activated human T-cells. ; FUNCTION: (Microbial infection) Acts as a receptor for Herpes simplex virus 1/HHV-1. ; FUNCTION: (Microbial infection) Acts as a receptor for Herpes simplex virus 2/HHV-2.
BTLA	B and T lymphocyte associated	Immunoinhibitor	Lymphocyte inhibitory receptor which inhibits lymphocytes during immune response.
PDCD1	Programmed cell death 1	Immunoinhibitor	Inhibitory cell surface receptor involved in the regulation of T-cell function during immunity and tolerance. Upon ligand binding, inhibits T-cell effector functions in an antigen-specific manner. Possible cell death inducer, in association with other factors.
HLA-DOB	Major histocompatibility complex, class II, DO beta	MHC	Important modulator in the HLA class II restricted antigen presentation pathway by interaction with the HLA-DM molecule in B-cells. Modifies peptide exchange activity of HLA-DM.
TAPBP	TAP binding protein (tapasin)	MHC	Involved in the association of MHC class I with transporter associated with antigen processing (TAP) and in the assembly of MHC class I with peptide (peptide loading).
CCL17	Chemokine (C-C motif)	Chemokine	Chemotactic factor for T-lymphocytes but not monocytes or granulocytes. May

	ligand 17		play a role in T-cell development in thymus and in trafficking and activation of mature T-cells. Binds to CCR4.
CCL19	Chemokine (C-C motif) ligand 19	Chemokine	May play a role not only in inflammatory and immunological responses but also in normal lymphocyte recirculation and homing. May play an important role in trafficking of T-cells in thymus, and T-cell and B-cell migration to secondary lymphoid organs.
XCL2	Chemokine (C motif) ligand 2	Chemokine	Chemotactic activity for lymphocytes but not for monocytes or neutrophils.

Supplementary Table 4 GO and KEGG pathway analyses of 14 signature genes

	**Term description**	**False discovery rate**
**GO_BP**	CCR chemokine receptor binding	3.46e-09
	immune system process	4.98e-09
	cellular response to tumor necrosis factor	8.30e-09
	T cell costimulation	4.40e-08
	positive regulation of T cell activation	2.40E-07
**GO_CC**	intrinsic component of membrane	0.0063
	side of membrane	0.0072
	external side of plasma membrane	0.0114
	intrinsic component of plasma membrane	0.0114
	plasma membrane	0.0114
**GO_MF**	CCR chemokine receptor binding	0.00012
	chemokine activity	0.00012
	signaling receptor activity	0.00012
	MHC protein complex binding	0.00031
	MHC class I protein binding	0.0011
**KEGG**	Cytokine-cytokine receptor interaction	8.32e-09
	Intestinal immune network for IgA production	6.95e-05
	Antigen processing and presentation	0.00015
	Graft-versus-host disease	0.0021
	Chemokine signaling pathway	0.0021
	Natural killer cell mediated cytotoxicity	0.0178
	Cell adhesion molecules (CAMs)	0.0190
	Herpes simplex infection	0.0275

FDR <0.05 was considered statistically significant. GO: Gene Ontology; KEGG: Kyoto Encyclopedia of Genes and Genomes; BP: biological processes; CC: cellular components; MF: molecular function; FDR: false discovery rate.
